# Efficient Production of Solar Hydrogen Peroxide Using Piezoelectric Polarization and Photoinduced Charge Transfer of Nanopiezoelectrics Sensitized by Carbon Quantum Dots

**DOI:** 10.1002/advs.202105792

**Published:** 2022-04-22

**Authors:** Xiaofeng Zhou, Fei Yan, Alexander Lyubartsev, Bo Shen, Jiwei Zhai, José C. Conesa, Niklas Hedin

**Affiliations:** ^1^ Shanghai Key Laboratory for R&D and Application of Metallic Functional Materials Functional Materials Research Laboratory School of Materials Science and Engineering Tongji University Shanghai 201804 China; ^2^ Department of Materials and Environmental Chemistry Stockholm University Stockholm SE 106 91 Sweden; ^3^ Institute of Catalysis and Petrochemistry CSIC Marie Curie 2 Cantoblanco Madrid 28049 Spain

**Keywords:** carbon quantum dots, hydrogen peroxide, Nb‐doped BaTiO_3_, piezocatalysis, piezoelectric polarization, piezophotocatalysis

## Abstract

Piezoelectric semiconductors have emerged as redox catalysts, and challenges include effective conversion of mechanical energy to piezoelectric polarization and achieving high catalytic activity. The catalytic activity can be enhanced by simultaneous irradiation of ultrasound and light, but the existing piezoelectric semiconductors have trouble absorbing visible light. A piezoelectric catalyst is designed and tested for the generation of hydrogen peroxide (H_2_O_2_). It is based on Nb‐doped tetragonal BaTiO_3_ (BaTiO_3_:Nb) and is sensitized by carbon quantum dots (CDs). The photosensitizer injects electrons into the conduction band of the semiconductor, while the piezoelectric polarization directed electrons to the semiconductor surface, allowing for a high‐rate generation of H_2_O_2_. The piezoelectric polarization field restricts the recombination of photoinduced electron–hole pairs. A production rate of 1360 µmol g_catalyst_^−1^ h^−1^ of H_2_O_2_ is achieved under visible light and ultrasound co‐irradiation. Individual piezo‐ and photocatalysis yielded lower production rates. Furthermore, the CDs enhance the piezocatalytic activity of the BaTiO_3_:Nb. It is noted that moderating the piezoelectricity of BaTiO_3_:Nb via microstructure modulation influences the piezophotocatalytic activity. This work shows a new methodology for synthesizing H_2_O_2_ by using visible light and mechanical energy.

## Graphical abstract

Carbon quantum dots sensitized Nb‐doped tetragonal BaTiO_3_ nanopiezoelectrics are considerably effective in simultaneously utilizing visible light and vibration energy, thus achieving high hydrogen peroxide yields from ethanol and water suspensions. This is because the sensitized piezoelectrics‐mediated polarization field accelerates the migration of photoinduced charge carriers.

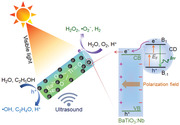

## 1 Introduction

As an efficient oxidant, hydrogen peroxide (H_2_O_2_) is frequently used in paper bleaching, textile processing, wastewater treatment, organic synthesis, and disinfection,^[^ ^1^ ^]^ including inactivating of the coronavirus SARS‐CoV‐2, which causes the COVID‐19 disease.^[^ ^2^ ^]^ H_2_O_2_ is also an H_2_‐rich energy carrier that can be stored, is soluble in water, and can be used to power direct peroxide fuel cells.^[^ ^3^ ^]^ The global market for H_2_O_2_ is expected to exceed 6 million metric tons by 2022.^[^ ^2^ ^]^ Traditionally, H_2_O_2_ is produced industrially by oxidation of anthraquinone, but the method has environmental problems. Large amounts of solid waste and wastewater are generated in the processes involving high‐pressure H_2_ produced from CH_4_.^[^ ^4^ ^]^ The production of H_2_O_2_ directly from mixtures of O_2_ and H_2_ has not yet been commercialized due to risks of explosion.^[^ ^5^ ^]^ So, new methods to synthesize H_2_O_2_ in a safe, cost‐effective, and sustainable manner are needed.

Using semiconductor‐based photocatalysis to convert H_2_O and O_2_ into H_2_O_2_ is promising for the industrial production of H_2_O_2_.^[^ ^1^, ^6^ ^]^ In this approach, photoinduced holes in the valance band of the semiconductor can oxidize H_2_O and generate O_2_; while electrons in the conduction band promote the two‐electron reduction of O_2_ and the generation of H_2_O_2_.^[^ ^7^ ^]^ Under favorable conditions, these redox reactions can produce H_2_O_2_ from H_2_O and O_2_ photocatalytically with sunlight at low gas pressures and ambient temperature. The associated sum reaction has a positive free‐energy change. Many semiconductor photocatalysts have been developed for this reaction but have been shown to produce only small amounts of H_2_O_2_ because the reaction kinetics are slow. For example, with a photocatalyst based on TiO_2_, the concentration of H_2_O_2_ was <0.5 mm after 24 h, which was not satisfactory.^[^ ^8^ ^]^ Although several surface‐modification techniques, including surface fluorination^[^ ^9^ ^]^ and surface complexation,^[^ ^10^ ^]^ have been studied to improve photocatalytic generation of H_2_O_2_ in TiO_2_‐based photocatalytic systems, the reactivity has not been significantly improved. The photoelectrochemical catalytic systems also have problems with low conversion efficiency at the photoelectrodes. In some cases, however, rapid H_2_O_2_ generation has been observed. With a 0.6 cm^2^ NiO photocathode in KCl aqueous solutions under irradiation with light having a wavelength of 623 nm, an H_2_O_2_ generation rate of 634 mmol g_photosensitizer_^−1^ h^−1^ was observed; a meso‐tetra (4‐carboxyphenyl) porphyrin dye was used for the sensitization.^[^ ^11^ ^]^ On the other hand, with organic photocatalysts such as resorcinol‐formaldehyde resins, an H_2_O_2_ production rate of 52 µmol g_catalyst_^−1^ h^−1^ was observed for photocatalysis.^[^ ^7b^ ^]^ This low rate seems to relate to aspects of the organic origin of this class of photocatalysts. New semiconductors may be required for efficient and photocatalytically induced H_2_O_2_ synthesis. It seems important to regulate the photoinduced charge‐transfer processes that can accelerate both the H_2_O oxidation and the selective two‐electron reduction of O_2_.

Most recently, piezoelectrics have been used to catalyze redox reactions for water splitting,^[^ ^12^ ^]^ organic synthesis,^[^ ^13^ ^]^ dye degradation,^[^ ^14^ ^]^ and H_2_O_2_ production.^[^ ^15^ ^]^ The typical piezocatalysts are noncentrosymmetric crystals, such as BaTiO_3_,^[^ ^16^ ^]^ BiFeO_3_,^[^ ^17^ ^]^ and ZnSnO_3_.^[^ ^18^ ^]^ The reaction rates of piezocatalysis are still far from being sufficiently high. This deficiency is primarily due to the periodic mechanical force being applied and the related piezopotentials failing to initiate the required critical Gibbs free energy threshold for redox reactions.^[^ ^19^ ^]^ In other words, the intensity of the piezoelectric polarization generated by the ultrasonic agitation is too low, because of limited charge carrier concentrations and the high impedances of these piezoelectrics. To address these problems, significant attempts have been made to improve the piezoelectricities of piezoelectrics. Methods such as control of the morphologies,^[^ ^13^, ^20^ ^]^ doping with metal ions,^[^ ^21^ ^]^ constructing heterostructures,^[^ ^22^ ^]^ and introducing defects^[^ ^13^, ^18^ ^]^ have been used. However, so far, rate enhancements with piezocatalysis are generally unsatisfactory, often being worse than with photocatalysis; hence, more efficient piezocatalysts need to be developed.

Although numerous efforts have been made to improve the reactivities of photocatalytic semiconductors such as the tuning of charge transfer^[^ ^23^ ^]^ and active sites,^[^ ^24^ ^]^ there is still a lot of room for improvements. Such development may promote a transition from related basic research to applied research. Coupling of piezoelectric and photoelectric effects in piezoelectric semiconductors is a proven and powerful approach that can enhance the catalytic activities for a variety of solar‐driven reactions.^[^ ^25^ ^]^ When a piezoelectric semiconductor is simultaneously subjected to mechanical energy and light irradiation, the generated piezopotential can modulate the migration and separation of internal photoinduced charge carriers, enhancing the catalytic activities in comparison to photocatalysis without piezoelectric polarization.^[^ ^26^ ^]^ For instance, an exceptional evolution rate of H_2_ of 12.2 mmol g_catalyst_^−1^ h^−1^ was achieved with g‐C_3_N_4_ nanosheets under visible light together with ultrasound irradiation, much above that of photocatalysis. The highest rate of H_2_O_2_ evolution obtained with ultrathin g‐C_3_N_4_ was 4.6 mmol g_catalyst_^−1^ h^−1^ in the piezophotocatalysis, which was 4.3 times that with bulk g‐C_3_N_4_ (1.1 mmol g_catalyst_^−1^ h^−1^) under identical reaction conditions.^[^ ^13a^ ^]^ This enhancement was related to the fact that anomalous piezoelectricity in ultrathin g‐C_3_N_4_ provided a powerful electrochemical driving force for the reduction reaction of H_2_O. Similarly, the piezoelectric Bi_4_NbO_8_Br showed an evolution rate of H_2_O_2_ of 792 µmol g_catalyst_^−1^ h^−1^ during piezophotocatalysis, which was substantially higher than that with piezo‐ and photo‐catalysis.^[^ ^27^ ^]^ These results revealed that the piezoelectric polarization improved the utilization of photoinduced electrons and holes in photoresponsive piezoelectric semiconductors. However, not all piezoelectric semiconductors exhibit enhanced photocatalytic performances under ultrasound activation, because the thermodynamic conditions of the piezophotocatalytic reactions must be met. The energy difference between conduction band electrons and valance band holes should exceed the targeted redox reaction barrier. It can be seen that the rates of water splitting into H_2_ and O_2_ with blue‐colored tetragonal BaTiO_3_ (*b*‐BaTiO_3_) nanoparticles in piezophotocatalysis were lower than those in piezocatalysis, which resulted from the insufficient redox potential of the conduction band and valance band of *b*‐BaTiO_3_; in this case, the piezoelectric polarization field was screened by the surface adsorption of H^+^/OH^−^ species.^[^ ^28^ ^]^ This means that the valance band potential must be higher than the oxidation potential of H_2_O to catalyze H_2_O oxidation during piezophotocatalysis. For catalyzed H_2_O reduction, the conduction band potential must be lower than the reduction potential of H^+^ cations. The general rule of piezocatalysis is that the generated piezopotential must reach the energy required for the redox reactions. Thus, at least in part, the energy‐band structure of the piezoelectric semiconductor determines its piezoelectric photocatalytic efficiency.

In general, common piezoelectrics with a perovskite structure have wide bandgaps.^[^ ^29^ ^]^ They have a generic chemical formula of ABO_3_. This wideness is related to the large difference in electronegativity of the O atom and the transition‐metal atom at the B site. In most piezoelectric materials of the perovskite type, the transition‐metal ion at the B site also plays a crucial role for the piezoelectricity.^[^ ^30^ ^]^ Owing to its excellent piezoelectric properties, BaTiO_3_ is one of the most studied piezocatalysts.^[^ ^31^ ^]^ The wide bandgap for BaTiO_3_ (3.2 eV) is typical for piezoelectric materials of the perovskite type, and the width is the single‐most significant drawback if visible light should be used. The wide bandgap is a consequence of the 3d° configuration of Ti^4+^, or its Ar‐like electronic structure. The piezoelectricity of BaTiO_3_ has been linked to its “d^0^”‐ness, which means that efforts to narrow the bandgap by substitution of Ti^4+^ with transition‐metal ions with oxidation states other than +4 usually results in the loss of piezoelectricity. Although codoping of bulk BaTiO_3_ with Mn and Nb cations has been shown to reduce the bandgap to 1.66 eV with a simultaneous 30% reduction of the intensity of the piezoelectric polarization, the related photocatalysis has not been thoroughly studied.^[^ ^32^ ^]^ In addition, dopants in the form of direct donors (such as Nb^5+^) can introduce charge compensation in doped BaTiO_3_ and thus affect the semiconductivity.^[^ ^33^ ^]^ This means that the carrier concentration (such as oxygen vacancies and titanium vacancies) of BaTiO_3_ can be altered by incorporating Nb into the crystal lattice of BaTiO_3_. As shown, moderate concentrations of dopants are usually helpful when it comes to improving the piezo‐ and piezophoto‐catalytic activities. For instance, nanowires of ZnSnO_3_ with −5.5 × 10^14^ cm^−3^ of oxygen vacancies had a longer carrier lifetime and produced more H_2_ than pristine ZnSnO_3_, although the oxygen vacancies deteriorated the ferroelectricity and piezoelectricity of ZnSnO_3_.^[^ ^18^ ^]^ The ZnSnO_3_ was of the R3c LiNbO_3_ type and the highest H_2_ production rate observed was 3450 µmol g^−1^ h^−1^. However, there are only few studies on catalytic H_2_O_2_ production with Nb‐doped piezoelectrics under the piezophototronic effect.

When carbon‐based nanoparticles^[^ ^34^ ^]^ are 2–10 nm in size and contain amorphous carbon and nanocrystalline regions of sp^2^‐hybridized graphitic carbon,^[^ ^35^ ^]^ they have strong optical absorption in the near‐visible region. While these carbon quantum dots (CDs) have light‐harvesting and electron‐transfer properties, they have been underexplored in photocatalysis and are used nearly exclusively as photosensitizers and for the photodegradation of organic dyes.^[^ ^36^ ^]^ However, the use of CDs as a primary photosensitizer has been studied for the photoreduction of CO_2_ to formic acid; CDs that had been passivated by poly(ethylene glycol) (PEG) and modified with deposits of the noble metals Au or Pt were used in that study.^[^ ^37^ ^]^

This study uses Nb‐doped BaTiO_3_ (BaTiO_3_:Nb) with CD photosensitizers to demonstrate piezophotocatalytic generation of H_2_O_2_ under visible light and ultrasound co‐irradiation. The CDs adhered to the BaTiO_3_:Nb and absorbed visible light at wavelengths >420 nm and injected photoinduced electrons into the conduction band of BaTiO_3_:Nb, resulting in a significant rise in the carrier concentration in the BaTiO_3_:Nb. Meanwhile, the ultrasound induced a piezoelectric polarization field in the BaTiO_3_:Nb, which accelerated the migration and separation of charge carriers. Based on a synergistic coupling of the piezoelectric polarization and photocatalysis, an H_2_O_2_ production rate of 1360 µmol h^−1^ g_catalyst_^−1^ was observed for the BaTiO_3_:Nb modified with CDs. This rate was observed in an ethanol–water solvent under simultaneous ultrasound and visible light irradiation, and with 0.14% of solar‐to‐chemical conversion (SCC) efficiency in 120 min of piezophotoreaction. Additionally, the CDs enhanced the conductivity of the BaTiO_3_:Nb and the rate of polarized charge transfer, which is of importance to piezocatalysis. A high production rate was also observed for piezocatalysis using the same catalyst. The anisotropic structure of the tetragonal BaTiO_3_:Nb nanorods had a higher carrier concentration compared to the pristine BaTiO_3_ nanoparticles, and in turn, the catalytic activities were improved for these nanorods when it came to the solar production of H_2_O_2_.

## 2 Results and Discussion

**Figure 1:**
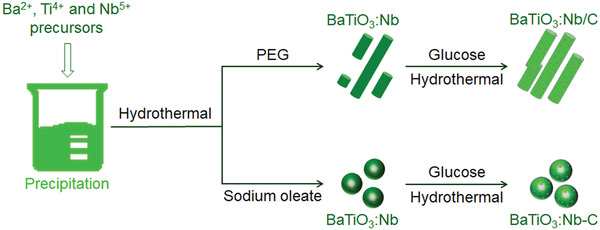
Schematic preparation processes for BaTiO_3_:Nb with different morphologies and modified with carbon quantum dots.

The piezophotocatalysts were prepared in two different ways, as shown in **Figure** **1**. The first step was to prepare BaTiO_3_:Nb hydrothermally together with the addition of PEG or sodium oleate. With PEG added, the BaTiO_3_:Nb had a nanorod‐like morphology, whereas with sodium oleate it had a spherical form. CDs were attached to the surface of BaTiO_3_:Nb using wet pyrolysis of an aqueous glucose solution.^[^ ^38^ ^]^ BaTiO_3_:Nb/C and BaTiO_3_:Nb‐C denote nanorod‐like and spherical shapes, respectively.

The microstructures of the BaTiO_3_‐based nanocomposites were investigated by electron microscopy. The BaTiO_3_ prepared with PEG added had a mixed structure with 80% nanorods and 20% nanoparticles (Figure S1a, Supporting Information), whereas the BaTiO_3_:Nb (also prepared with PEG added) with an average diameter of ≈200 nm had 95% nanorods and 5% nanoparticles (Figure S1b, Supporting Information). How to prepare uniform BaTiO_3_ and BaTiO_3_:Nb nanorods with the same aspect ratio by hydrothermal methods are worth investigating in follow‐up studies. The diameters of the nanorods in BaTiO_3_:Nb/C (Figure S1c,d, Supporting Information) were somewhat larger than for BaTiO_3_:Nb and ascribed to ripening during the second hydrothermal process, in a similar manner as the dimensions of the nanorods in BaTiO_3_:Nb was larger than that of pristine BaTiO_3_. BaTiO_3_:Nb‐C prepared with sodium oleate had spherical nanoparticles with an average diameter of ≈80 nm (Figure S1e,f, Supporting Information). The CDs were very small for both BaTiO_3_:Nb/C and BaTiO_3_:Nb‐C and did not affect the overall morphologies of the composite nanoparticles. The sample prepared by wet pyrolysis of an aqueous glucose solution^[^ ^39^ ^]^ contained hydrochar particles that were larger (300–450 nm, see in Figure S2, Supporting Information) than for the CDs prepared with BaTiO_3_:Nb. The reduced size of the CDs in these latter preparations was related to nucleation sites on the BaTiO_3_:Nb or its catalytic effects. The CDs also had a comparably condensed nature.

**Figure 2:**
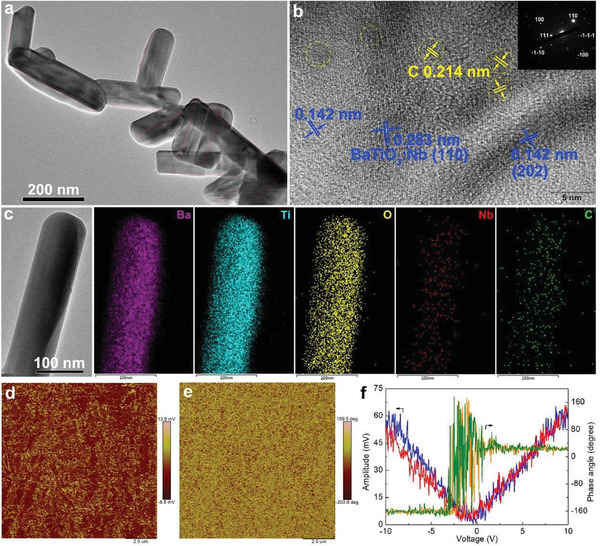
Structures of BaTiO_3_:Nb/C. a) A transmission electron microscopy image and b) a high‐resolution TEM image of BaTiO_3_:Nb/C and a corresponding selected area electron diffraction pattern (inset). c) Scanning electron microscopy energy‐dispersive X‐ray spectroscopic elemental mapping images of BaTiO_3_:Nb/C. The out‐of‐plane piezoresponse force microscope images of BaTiO_3_:Nb/C for d) amplitude, e) phase, and f) piezoelectric hysteresis loop at “off” state.

The length of the nanorods in BaTiO_3_:Nb/C was in the order of tens and hundreds of nanometers as can be seen from the transmission electron microscopy (TEM) image in **Figure** **2**a. Features in the high‐resolution TEM (HRTEM) image of BaTiO_3_:Nb/C in Figure 2b show that the sample had a high crystallinity with lattice fringes of 0.283 and 0.142 nm, which were assigned to the (110) and (202) facets of BaTiO_3_:Nb. The inset of Figure 2b shows the selected‐area electron diffraction (SAED) pattern from a single‐crystalline BaTiO_3_:Nb particle. The lattice spacing of the carbon dot (which has been highlighted by a bright yellow circle) in the HRTEM image was 0.214 nm, which is similar to the hexagonal pattern of graphene with d_1100_.^[^ ^40^ ^]^ It is still an open question why and if CDs prepared under wet pyrolysis have a crystalline nature;^[^ ^41^ ^]^ here it seems as if those catalyzed/promoted by the BaTiO_3_:Nb/C were crystalline. The dispersion of the elements in BaTiO_3_:Nb/C was homogenous at the resolution of the energy dispersive X‐ray spectroscopy (EDS) used (Figure 2c). Overall, it can be concluded that the CDs with an average size of 3 nm had been successfully loaded onto the surface of BaTiO_3_:Nb by wet pyrolysis of an aqueous glucose solution.

Representative amplitude and phase images of BaTiO_3_:Nb/C from piezoresponse force microscopy (PFM) are shown in Figure 2d,e. The amplitude and phase signals of BaTiO_3_:Nb/C had a clear contrast, indicating that BaTiO_3_:Nb/C had robust ferroelectricity and piezoelectricity. The phase‐voltage curve had a 180° change under a 10 V DC bias (Figure 2f), revealing a ferroelectric polarization switching in the BaTiO_3_:Nb/C. The V‐shaped hysteresis loop from the amplitude–voltage curve further confirmed an excellent piezoelectric response. Similar results were observed for BaTiO_3_ and BaTiO_3_:Nb (Figure S3, Supporting Information), suggesting that the ferroelectricity and piezoelectricity of BaTiO_3_:Nb/C were not affected by the Nb doping and depositing of the CDs, and that the significant piezoelectric polarization fields were generated as responses to the mechanical vibration. Such polarization fields improve the separation and transfer of photoinduced charge carriers. It is worth noting that the piezoelectricity of BaTiO_3_:Nb slightly deteriorated on increasing the Nb doping levels. The piezoelectric coefficient of BaTiO_3_:Nb was 0.32 pm V^−1^ lower than that of BaTiO_3_. It was tentatively ascribed to the “d^0^”‐ness of the BaTiO_3_.^[^ ^32^ ^]^

**Figure 3:**
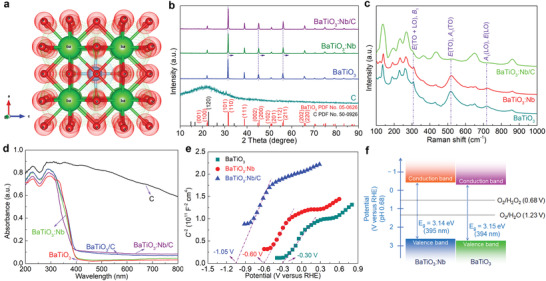
Structural and electronic properties of various catalysts. a) Crystal structure of tetragonal BaTiO_3_. b) X‐ray diffraction patterns of pure carbon, BaTiO_3_, BaTiO_3_:Nb, and BaTiO_3_:Nb/C. c) Raman spectra of BaTiO_3_, BaTiO_3_:Nb, and BaTiO_3_:Nb/C. d) Diffuse reflectance ultraviolet–visible absorption spectra of the catalysts. e) Electrochemical Mott–Schottky curves of BaTiO_3_, BaTiO_3_:Nb, and BaTiO_3_:Nb/C. f) Band structures of BaTiO_3_ and BaTiO_3_:Nb.

The crystal structure of the pristine BaTiO_3_ is shown in **Figure** **3**a. It has a tetragonal structure, with Ti^4+^ off‐center in the TiO_6_ octahedra, resulting in a net polarization along the [001] edge direction. Analyses of the powder X‐ray diffraction (XRD) patterns in Figure 3b showed that both undoped and doped BaTiO_3_ belonged to a tetragonal phase of the space group *P*4*mm*(99).^[^ ^42^ ^]^ The half‐peak widths for the XRD lines were slightly wider for BaTiO_3_:Nb/C and BaTiO_3_:Nb than for BaTiO_3_ (Figure S4, Supporting Information). The tetragonal (101)/(110) diffraction peaks of BaTiO_3_:Nb/C and BaTiO_3_:Nb shifted to higher angles (about 0.12° shifts) when compared to pristine BaTiO_3_ (Figure S4, Supporting Information) as a result of Nb^5+/4+^ (0.064/0.068 nm) replacing in part larger Ba^2+^ (0.135 nm), according to the analysis using the Bragg equatio, 2*d*sin*θ* = *λ*. The decreasing unit cell volume and crystal lattice thus altered the internal strain of the crystals. Similar A‐site substitution doping was found in a study of Nb‐doped SrTiO_3_ films, and the strain in the films was initiated by rod‐type Sr vacancy clusters, resulting in an improved electron mobility with values from 7652 to 53 000 cm^2^ V^−1^ s^−1^ at 2 K.^[^ ^43^ ^]^ The carrier concentration of the BaTiO_3_:Nb discussed below was higher than that of undoped BaTiO_3_, verifying that the crystal engineering approach of this study was effective in enhancing the electron mobility while simultaneously maintaining the piezoelectricity. The low contents of CDs in the BaTiO_3_:Nb/C caused its XRD pattern to be almost identical to that of BaTiO_3_:Nb. The pure carbon (hydrochar, C) had a wide X‐ray scattering peak, as shown in Figure 3b. Hydrochars prepared from carbohydrates are known to have a polymeric nature and a high carbon content.^[^ ^44^ ^]^ The Raman spectra of BaTiO_3_, BaTiO_3_:Nb, and BaTiO_3_:Nb/C are shown in Figure 3c. The E(TO + LO), B1 peak at 305 cm^−1^, E(TO), A_1_(TO) peak at 516 cm^−1^, and the A_1_(LO), E(LO) peak at 716 cm^−1^ are specific to noncentrosymmetric tetragonal BaTiO_3_.^[^ ^45^ ^]^ This finding implies that the Nb doping and modification with CDs had no major effect on the phase structure of BaTiO_3_.

The diffuse reflectance ultraviolet–visible (UV–vis) spectra of the hydrochar, BaTiO_3_, BaTiO_3_:Nb, BaTiO_3_/C, and BaTiO_3_:Nb/C are shown in Figure 3d. The black pure carbon had the highest visible light absorption. BaTiO_3_:Nb had a similar absorption edge (around 380 nm) as BaTiO_3_, but due to its doped nature, it had higher visible light absorption. The BaTiO_3_:Nb/C and BaTiO_3_/C had higher absorption than the related compounds without CDs. Thus, the high c generation rates of H_2_O_2_ during photo‐ and piezophoto‐catalysis for BaTiO3:Nb/C were tentatively ascribed to the CD‐related visible light absorption. For the BaTiO_3_‐based catalysts (Figure S5, Supporting Information), the bandgaps were reduced on the deposition of CDs.

The electrochemical Mott–Schottky curves (Figure 3e and Figure S6, Supporting Information) show the n‐type semiconducting features of the samples. Moreover, the flat‐band potentials (*E*_FB_) of the BaTiO_3_:Nb and BaTiO_3_ were, respectively, determined to be −0.38 and −0.30 V versus the reversible hydrogen electrode (RHE) in Na_2_SO_4_ aqueous solution at pH = 6.8. The band edge positions are calculated according to the following Equations (1) and (2):

(1) $$E_{\mathrm{CB}}=E_{\mathrm{FB}}\pm 0.05V$$

(2) $$E_{\mathrm{VB}}=E{}_{g}^{\prime }+E_{\mathrm{CB}}$$

where *E*_CB_ and *E*_VB_ are the conduction band and valance band edge potentials, respectively. *E*′_g_ is the absolute value of the bandgap energy, that is, *E*_g_/eV. Hence, the *E*_CB_ of BaTiO_3_:Nb was estimated to lie between −0.43 and −0.33 V, and for BaTiO_3_ between −0.35 and −0.25 V versus RHE given a potential difference of 0–1 V between the *E*_FB_ and the *E*_CB_ in n‐type semiconductors. From the *E*_g_, the *E*_VB_ of BaTiO_3_:Nb and BaTiO_3_ were estimated to be 2.88–2.96 V and 2.91–3.01 V versus RHE, respectively. It is clear that both BaTiO_3_:Nb and BaTiO_3_ possess thermodynamic driving forces for the two‐electron reduction of O_2_ to H_2_O_2_. The proposed band structures of BaTiO_3_ and BaTiO_3_:Nb are shown in Figure 3f.

The valance bands of both BaTiO_3_ and BaTiO_3_:Nb were derived from analyses of X‐ray photoelectron spectroscopy (XPS) valance spectra shown in Figure S7, Supporting Information. BaTiO_3_:Nb displayed a valance band with the edge of the maximum energy at about 1.80 eV, while the valance band maximum energy of the BaTiO_3_:Nb/C was upshifted by 0.18–1.62 eV compared to BaTiO_3_. From the point of view of kinetic and thermodynamic requirements for direct photocatalytic reactions on semiconductors with photoexcitation, features in the width of the valance band and the minimum energy of the conduction band were noted. A wider valance band promotes the separation of charge carriers, as the width intrinsically enhanced the mobility of the holes. Consequently, a wider valance band causes more photo‐oxidation of holes. The upshifting of the conduction band should play two crucial roles for photocatalysis. An increase in the conduction band minimum makes photogenerated electrons more prone to react with O_2_ to generate •O_2_^−^ and promotes transfer of photoinduced electrons to the reactants, inhibiting the recombination of photoinduced electron–hole pairs. The valance band XPS spectrum indicated metallic properties of BaTiO_3_:Nb/C, originating from the C 1s state passing through the Fermi level (0 eV). This finding validated a small or absent energy gap for the CSs and their occupied and unoccupied levels; however, they could produce electron–hole pairs by interband transitions.^[^ ^46^ ^]^

The chemical states and bonding structures of the samples were studied with XPS. From the XPS survey spectra (Figure S8a, Supporting Information) it was concluded that all samples had predicted characteristic bands. The high‐resolution XPS spectra from high to low binding energies are given in Figure S8b–f, Supporting Information. Analyses of the Ba 3d_5/2_ peaks suggested that the Ba ions existed in the form of an ABO_3_ perovskite structure (which corresponded to the binding energies of 778.5 and 793.8 eV) and a nonperovskite structure (which corresponded to the binding energies of 779.5 and 794.8 eV). Ba 3d_5/2_ peaks were observed with binding energies of 779.5 and 778.5 eV and Ba 3d_3/2_ peaks at the binding energies of 794.8 and 793.8 eV (Figure S8b, Supporting Information). The Ti 2p XPS spectrum (Figure S8c, Supporting Information) of BaTiO_3_:Nb exhibited an ≈0.2 eV blueshift relative to the BaTiO_3_, suggesting that the Nb doping had affected the Ti—O bond. However, the Ti 2p XPS spectrum of BaTiO_3_:Nb/C had an ≈0.4 eV redshift relative to the one of BaTiO_3_:Nb, which might be attributed to the Ti—O bonds being distorted during the hydrothermal treatment. There were no noticeable energy shifts of the O 1s peaks for BaTiO_3_, BaTiO_3_:Nb, and BaTiO_3_:Nb/C, suggesting identical oxygen chemical states in BaTiO_3_ and BaTiO_3_:Nb. Notably, the peak at ≈532.0 eV was assigned to the OH;^[^ ^47^ ^]^ in BaTiO_3_:Nb/C, the fingerprints of protonated hydroxide groups H+OH (at 532.6–532.9 eV) was ascribed to sideway coordination of an HOH site on the oxygen lattice,^[^ ^48^ ^]^ which typically arises from ambient moisture adsorption on the BaTiO_3_:Nb/C surface. Such moieties are stable even under a high vacuum. From an analysis of the Nb 3d XPS spectrum (Figure S8e, Supporting Information), the Nb cations could exist as Nb_2_O_5_ and/or NbO_2_, confirming that Nb bonded with O in BaTiO_3_:Nb and BaTiO_3_:Nb/C. Peaks in the C 1s XPS spectrum (Figure S8f, Supporting Information) for the BaTiO_3_:Nb/C were assigned to four bands for C═C, C—C, C—O—C, and O—C═O bonds, implying that the CDs had a characteristic of graphitic carbon,^[^ ^49^ ^]^ which was supported by the TEM image in Figure 2b. These results suggest a successful modification of BaTiO_3_:Nb by CDs with graphite features even though the temperature for the wet pyrolysis was low.

Photoelectrochemical characterizations were performed to further support the analysis of the high rate of H_2_O_2_ generation observed with BaTiO_3_:Nb/C. Time‐resolved photoluminescence (PL) spectra of bulk BaTiO_3_:Nb and BaTiO_3_:Nb/C are presented in Figure S9a, Supporting Information. These spectra probe the specific charge carrier dynamics of the studied nanosystems. The average emission lifetime of BaTiO_3_:Nb/C was 0.96 ns, which was slightly shorter than that of BaTiO_3_:Nb (1.00 ns). Meanwhile, the intensity of the steady‐state PL peak for BaTiO_3_:Nb/C was much lower than for BaTiO_3_:Nb (Figure S9b, Supporting Information), indicating a significant PL quenching and lower recombination rate of the photogenerated electron–hole pairs with BaTiO_3_:Nb/C. The PL quenching and lifetime decrease indicate the formation of an electron transfer from CDs to BaTiO_3_:Nb occurring in a nonradiative quenching manner. Efficient interfacial charge migration and separation were achieved in BaTiO_3_:Nb/C. Features in the transient photocurrent spectra clearly showed that the BaTiO_3_:Nb/C had an enhanced photocurrent compared with BaTiO_3_:Nb and BaTiO_3_ (Figure S9c, Supporting Information), revealing promotion and transfer of photogenerated electron–hole pairs in BaTiO_3_:Nb/C. However, the electrochemical impedance spectrum (EIS) of BaTiO_3_:Nb/C had a smaller semicircle than its parent compounds, which can be seen from the Nyquist plots of Figure S9d, Supporting Information. This shrunken semicircle indicated a low charge‐transfer resistance in the BaTiO_3_:Nb/C allowing rapid transport and separation of photoinduced charges. The charge‐transfer resistance of BaTiO_3_:Nb was lower than for undoped BaTiO_3_, which showed an easier transfer of electrons. The pure carbon had the minimum charge‐transfer resistance among all studied samples, which was related to its electrical conductivity. All these observations demonstrated that the BaTiO_3_:Nb/C had enhanced generation, separation, and transport of photoinduced charges, and hence, a high efficiency of photocatalytic and piezo(photo)catalytic production of H_2_O_2_.

The piezo‐, photo‐, and piezophoto‐catalytic performances of BaTiO_3_:Nb/C and BaTiO_3_:Nb‐C were studied for the generation of H_2_O_2_. First, the effects of BaTiO_3_:Nb with nanorod‐like and spherical shapes were investigated. The H_2_O_2_ generation rates were substantially higher for nanorod‐like BaTiO_3_:Nb (Figure S10a–c, Supporting Information). Thus, further experimentation was carried out with nanorod‐like BaTiO_3_ unless otherwise noted. With respect to the Nb‐content, the piezophotocatalytic activities of BaTiO_3_:Nb, first increased and then decreased, and the ideal molar ratio of Nb:Ba was 1:100, as shown in Figure S10d, Supporting Information. As a result, this ratio was used for all subsequent BaTiO_3_:Nb samples.

**Figure 4:**
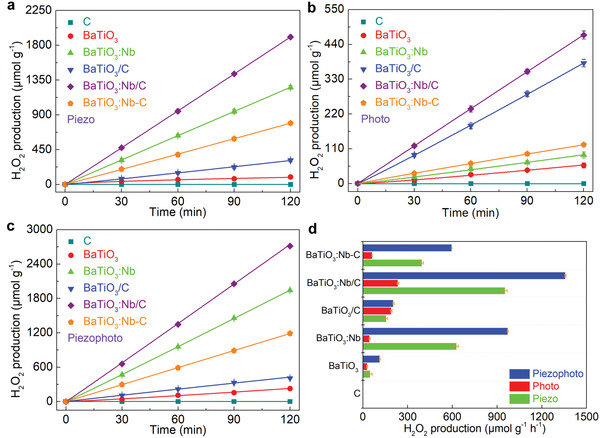
Catalytic performance of various catalysts for the production of H_2_O_2_. a) Time dependencies of the amount of H_2_O_2_ produced with various catalysts in an ethanol–water solvent under 40 kHz ultrasonic irradiation. b) Time dependencies of the amount of H_2_O_2_ formed with various catalysts in an ethanol–water solvent under visible light (*λ* > 420 nm) irradiation. c) Time dependencies of the amount of H_2_O_2_ formed with various catalysts in an ethanol–water solvent under simultaneous ultrasound and visible light irradiation. d) Comparison of the H_2_O_2_ production rates with various catalysts in piezo‐, photo‐, and piezophoto‐catalytic reactions. C denotes preparations with carbon quantum dots, /C in nanorod‐like nanoparticles, and ‐C in spherical nanoparticles.

Piezocatalytic H_2_O_2_ production rates (40 kHz, 150 W) are shown in **Figure** **4**a. High rates were observed with the BaTiO_3_‐based catalysts. The BaTiO_3_:Nb (628 µmol g_catalyst_^−1^ h^−1^) produced nearly 13 times as much H_2_O_2_ as the BaTiO_3_ (48 µmol g_catalyst_^−1^ h^−1^) because the carrier concentration of BaTiO_3_:Nb was higher than that of BaTiO_3_. Despite BaTiO_3_:Nb having a slightly smaller piezoelectricity than BaTiO_3_, the macroscopic polarization intensity was not significantly changed on the A‐site doped BaTiO_3_:Nb. Therefore, the main reason for the enhancement of the piezocatalytic activities of BaTiO_3_:Nb was that the Nb dopants increased the carrier concentration and reduced the charge transfer resistance. Accordingly, the rates of BaTiO_3_:Nb/C and BaTiO_3_/C increased to 953 and 156 µmol g_catalyst_^−1^ h^−1^, respectively, because the CDs facilitated the free charge transfer on the surfaces of BaTiO_3_:Nb and BaTiO_3_. This mechanism is the same as in a related study where Pd nanoparticles were used to promote the piezocatalytic H_2_ production rate of BiFeO_3_ nanosheets.^[^ ^17b^ ^]^ Moreover, the rate of H_2_O_2_ production for BaTiO_3_:Nb/C was 2.4 times higher than for BaTiO_3_:Nb‐C (397 µmol g_catalyst_^−1^ h^−1^). The enhanced rate was attributed to the anisotropic form of the BaTiO_3_:Nb nanoparticles and the higher piezopotential as compared to more isotropic particles under the same external force as discussed below in relation to a finite element analysis. This finding suggests that the size and shape of the particles affect the piezocatalytic activities. The size and shape have an effect on the piezopotential, which have been shown for piezoelectric materials such as monolayers of MoS_2_^[^ ^50^ ^]^ and GaN‐based nanowires.^[^ ^51^ ^]^ The reduced dispersibility of BaTiO_3_:Nb‐C in an ethanol–water solvent compared with BaTiO_3_:Nb/C (Figure S11a, Supporting Information) further weakened its piezocatalytic activity. This reduction was tentatively ascribed to a reduced effective contact area for less dispersed materials with respect to the reactants in the solution. Since the dispersion of particles was obtained under static conditions, the relationship between the water dispersibility of the catalyst and its catalytic activity will be further discussed below. In addition, the rate of H_2_O_2_ production with nanorod‐like BaTiO_3_:Nb was higher than with BaTiO_3_:Nb‐C, indicating that contribution from the piezopotential was greater than the CDs‐sensitization of the surface‐charge transfer. These results demonstrated that the piezoelectricity and conductivity are important for high‐performing piezocatalysts. For the pure carbon, no H_2_O_2_ production occurred, which was consistent with its inability to create a piezopotential. Only a very small amount of H_2_O_2_ production (4 µmol in 120 min) was observed in the control experiments with only an ethanol–water solution (Figure S12a, Supporting Information). The sonocatalytic effect^[^ ^52^ ^]^ induced by the ultrasound waves could therefore be neglected in this reaction system.

The photocatalytic effects were studied under visible light (*λ* > 420 nm) irradiation, and the production rates of H_2_O_2_ are shown in Figure 4b. Also with photocatalysis, the BaTiO_3_:Nb/C showed the highest production rate among the studied catalysts with a rate of 234 µmol g_catalyst_^−1^ h^−1^. The next highest rate was observed for the undoped BaTiO_3_/C with a value of 190 µmol g_catalyst_^−1^ h^−1^. The reduced rate for the undoped heterostructure was ascribed to the larger charge‐transfer resistance of BaTiO_3_/C than BaTiO_3_:Nb/C. For BaTiO_3_:Nb‐C with spherical morphologies, the rate for H_2_O_2_ production was significantly reduced (61 µmol g_catalyst_^−1^ h^−1^). This reduction suggested that the size and shape of the Nb‐doped BaTiO_3_ affected the photocatalytic performances. The mean value of the particle size distribution (Figure S11a, Supporting Information) for BaTiO_3_:Nb/C (399 nm) were smaller than for BaTiO_3_:Nb‐C (518 nm), which in turn means that the dispersibility of the BaTiO_3_:Nb/C was higher than for BaTiO_3_:Nb‐C in an ethanol–water solvent. Taking the SEM results into account, it was inferred that the small size effect of spherical BaTiO_3_:Nb‐C led to the difference in water dispersibilities between BaTiO_3_:Nb/C and BaTiO_3_:Nb‐C, because the agglomeration of BaTiO_3_:Nb‐C in ethanol–water solution decreased their own surface energies. This observation suggested that the photocatalytic activities of BaTiO_3_:Nb‐C can be further improved by surface modification and the regulation of solvent compositions. Although the generation rate of H_2_O_2_ for BaTiO_3_:Nb (45 µmol g_catalyst_^−1^ h^−1^) was 1.6 times that of undoped BaTiO_3_ (29 µmol g_catalyst_^−1^ h^−1^), the rates were much smaller than for BaTiO_3_:Nb/C and BaTiO_3_/C. This finding shows that the CDs played a critical role as visible light absorbers and improved the photocatalytic activities of the BaTiO_3_:Nb and BaTiO_3_. Note that the photocatalytic activities of BaTiO_3_:Nb and BaTiO_3_ related to their absorbances were not null in the visible light range. A similar result has been observed with NaNbO_3_ which has a wide bandgap (3.5 eV).^[^ ^53^ ^]^ With this photocatalyst, an H_2_ production rate of 0.3 µmol h^−1^ g^−1^ was observed with irradiation in the visible light (*λ* > 420 nm). The rate of H_2_O_2_ production with BaTiO_3_:Nb‐C was lower than with BaTiO_3_/C, suggesting that the nanorod structure of prestine BaTiO_3_ was more conducive to enhancing the photocatalytic activity than the spherical structure of doped BaTiO_3_:Nb. This difference was ascribed to the lower dispersibility of catalysts with spherical structures (BaTiO_3_:Nb‐C) (Figure S11a, Supporting Information) and thus a lower utilization ratio of the injected electrons under visible light irradiation. It was observed that the Nb doping, sensitization of CDs, and anisotropic structure of the piezoelectric semiconductor synergistically enhanced the rates of visible light driven H_2_O_2_ production. There was no H_2_O_2_ production in the ethanol–water solutions without catalysts (Figure S12a, Supporting Information), and with the pure carbon, almost no H_2_O_2_ production (Figure 4b) was observed.

It was surprising that the H_2_O_2_ production rates were significantly improved when ultrasound (40 kHz, 150 W) was simultaneously used with visible light (*λ* > 420 nm, 100 mW cm^−2^). This was true for all the BaTiO_3_‐based catalysts studied. As shown in Figure 4c, the different catalysts had the same ordering for piezophotocatalysis and piezocatalysis. In relation to photocatalysis, a slightly different ordering of the catalyst with respect to the catalytic effects was observed. This difference and the enhancement in piezophotocatalysis are related to the fact that the band structures of the semiconductors determine the thermodynamic conditions of piezophotocatalytic reactions. The efficiencies of the catalysts in converting mechanical energy into H_2_O_2_‐based chemical energy are higher than those of converting visible light energy into H_2_O_2_ energy for the present reaction system, and hence the overall activities are lower for photocatalysis than for piezo‐ and the piezophoto‐catalysis. The rates of H_2_O_2_ production for the BaTiO_3_‐based catalysts in piezophotocatalysis were also influenced by the anisotropic nanorod structures, Nb doping, and CDs loading. The fastest generation of H_2_O_2_ was achieved with BaTiO_3_:Nb/C (1360 µmol g_catalyst_^−1^ h^−1^). It was 1.4 and 3.2 times faster than with BaTiO_3_:Nb (972 µmol g_catalyst_^−1^ h^−1^) and BaTiO_3_/C (430 µmol g_catalyst_^−1^ h^−1^). This finding showed that the Nb doping was important to obtain the high piezophotocatalytic activities. The rate of H_2_O_2_ generation for BaTiO_3_:Nb‐C (1190 µmol g_catalyst_^−1^ h^−1^) was much lower than for BaTiO_3_:Nb/C, suggesting that the anisotropic particles accelerated the separation and transfer of photogenerated charges by the piezoelectric polarization. The piezophotocatalytic activities of BaTiO_3_:Nb were higher than for BaTiO_3_ and can be ascribed to a smaller impedance of the doped BaTiO_3_:Nb. In addition, the response of BaTiO_3_:Nb in the visible light region was also stronger than for BaTiO_3_. Note that the rate of H_2_O_2_ generation with BaTiO_3_:Nb/C via the piezophotocatalytic approach far exceeded that recently reported with zinc polyphthalocyanine decorated on photocatalysts based on boron‐doped carbon nitride^[^ ^54^ ^]^ (Table S1, Supporting Information). However, high rates of H_2_O_2_ generation under piezophotocatalysis have been observed with ultrathin g‐C_3_N_4_.^[^ ^12^ ^]^

When it came to catalytic activity, the BaTiO_3_:Nb had an optimum at intermediate CD loadings. The CDs were derived and deposited in situ during wet pyrolysis, and the optimal loading was achieved for a glucose concentration of 8 mmol/dm^3^. The catalysts derived under these conditions had the highest rate of H_2_O_2_ production (BaTiO_3_:Nb/C), see Figure S12b, Supporting Information. When the CD content was too low, the optical absorption seemed not sufficiently improved and when the CD content was too high, the CDs seemed to act as recombination centers and reduce the utilization ratio of the photoinduced electrons. Another aspect is that the piezophotocatalytic activity of BaTiO_3_:Nb/C decreased with decreasing visible light intensity (Figure S12c, Supporting Information), and the optimal ultrasonic power for the H_2_O_2_ generation was 150 W (Figure S12d, Supporting Information). The nonlinear dependency of the rate of H_2_O_2_ generation on the ultrasonic power and the linear dependency on the visible light intensity for BaTiO_3_:Nb/C indicated that there is a synergistic effect between the intensity of the piezoelectric polarization and the photogenerated charge transfer. These trends are different from the cooperation of indirect hot electron transfer and direct charge transfer initiated by localized surface plasmons in Ag_20_ and Ag_147_ nanoclusters leading to an efficient CO_2_ photoreduction in another study.^[^ ^55^ ^]^ In the systems studied here, the effective carrier concentration involved in the piezophotocatalysis was influenced by the piezophototronic effect. However, there is no specific mechanism to explain the effect of piezoelectric polarization on the kinetics of the piezophotocatalytic reactions, so further studies are needed to clarify potential generality. In addition, the piezophotocatalytic rates of H_2_O_2_ generation for BaTiO_3_:Nb/C and BaTiO_3_:Nb‐C were much higher in a mixed ethanol–water solvent than in pure ethanol or water. The optimimal volume ratio of ethanol:water was 1:9 (Figure S13, Supporting Information). This finding suggested that the ethanol effectively balanced the separation of electron–hole pairs and the utilization of electrons for the reduction of O_2_/H_2_O to H_2_O_2_. Furthermore, it was observed that the piezophotocatalytic rates of H_2_O_2_ generation for BaTiO_3_:Nb/C were always higher than for BaTiO_3_:Nb‐C under the same reaction conditions (in water, ethanol, or ethanol–water solutions), which further provided a solid proof that the anisotropic axial structure enhanced the piezophotocatalytic activities of the catalysts. Under visible light irradiation, the rate of H_2_ generation for BaTiO_3_:Nb/C was 239 µmol g_catalyst_^−1^ h^−1^ in an ethanol–water solvent (Figure S14, Supporting Information), while neither H_2_ nor O_2_ were observed with BaTiO_3_:Nb/C in pure water or ethanol. These findings show that the ethanol was a hole scavenger and prevented the recombination of photoinduced electrons and holes, thus improving the reaction rates critical for the formation of H_2_O_2_ and H_2_. Although the dispersibility of BaTiO_3_:Nb/C in ethanol–water solvents was reduced compared with pure ethanol and water (Figure S11b, Supporting Information), the mixed solvent was required to obtain highrates of H_2_O_2_ and H_2_ generation. The physical effect of the dispersibility had a small influence on the catalytic activity compared with the change of reaction kinetics for the generation of H_2_O_2_ and H_2_ via the involvement of ethanol. In other words, improving the water dispersibility of the catalysts must not affect the chemical enhancement of the reaction kinetics negatively in the present reaction system. The pure carbon (Figure 4c) did not produce any H_2_O_2_ during co‐irradiation with ultrasound and visible light, and the rate of H_2_O_2_ generation in the control experiment (Figure S12a, Supporting Information) was about 1% than that observed with BaTiO_3_:Nb/C. This means that the generation of H_2_O_2_ was initiated by the BaTiO_3_‐based catalysts during the piezophotocatalytic processes.

The rates of H_2_O_2_ generation are summarized in Figure 4d for the catalytic systems of this study, and the piezophotocatalytic reaction rates were higher than for the isolated piezo‐ and photo‐catalysis for all the BaTiO_3_‐based catalysts. It was also clear that sole piezocatalysis was superior to photocatalysis. This phenomenon can be attributed to the piezoelectric response of the BaTiO_3_‐based catalyst which is sensitive to visible light, which has been confirmed in studies of KNbO_3_ nanostructures.^[^ ^56^ ^]^ In this study, the BaTiO_3_:Nb/C induced the highest rate of H_2_O_2_ generation. Consistently, the rates of generation were enhanced by nanorod‐like particles and by modification with CDs. Unlike semiconductor photocatalysis (which relies on the band potential difference to initiate redox reactions), piezocatalysts accelerate redox reactions through piezopotentials. These are not limited by the inherent potential difference between the energy bands. As long as the piezopotential induced by the piezoelectric polarization field is higher than the reaction threshold, piezocatalytic reactions can occur at the surfaces of the piezoelectrics. This situation also means that the reaction selectivity of piezocatalysis can be moderated differently than in photocatalysis. In this study, the piezophotocatalytic activity was slightly higher for piezophotocatalysis than for piezocatalysis and can be related to the piezoelectric polarization field. This field is initiated by ultrasound and accelerates the separation and transportation of photoinduced charge carriers. Accordingly, as shown in Table S2, Supporting Information, the apparent quantum yield (*Φ*_AQY_) and SCC efficiency for piezophotocatalytic H_2_O_2_ production with BaTiO_3_:Nb/C were as high as 0.03% and 0.14%, which were much higher than for photocatalysis. In addition, the rate of H_2_O_2_ generation on BaTiO_3_:Nb/C remained identical to the initial ones after 12 h of recycling (Figure S15, Supporting Information). The SEM images of the BaTiO_3_:Nb/C before and after reaction (Figure S16, Supporting Information) showed that its surface structure did not change, suggesting that the BaTiO_3_:Nb/C had excellent stability during the piezophotocatalytic reactions.

**Figure 5:**
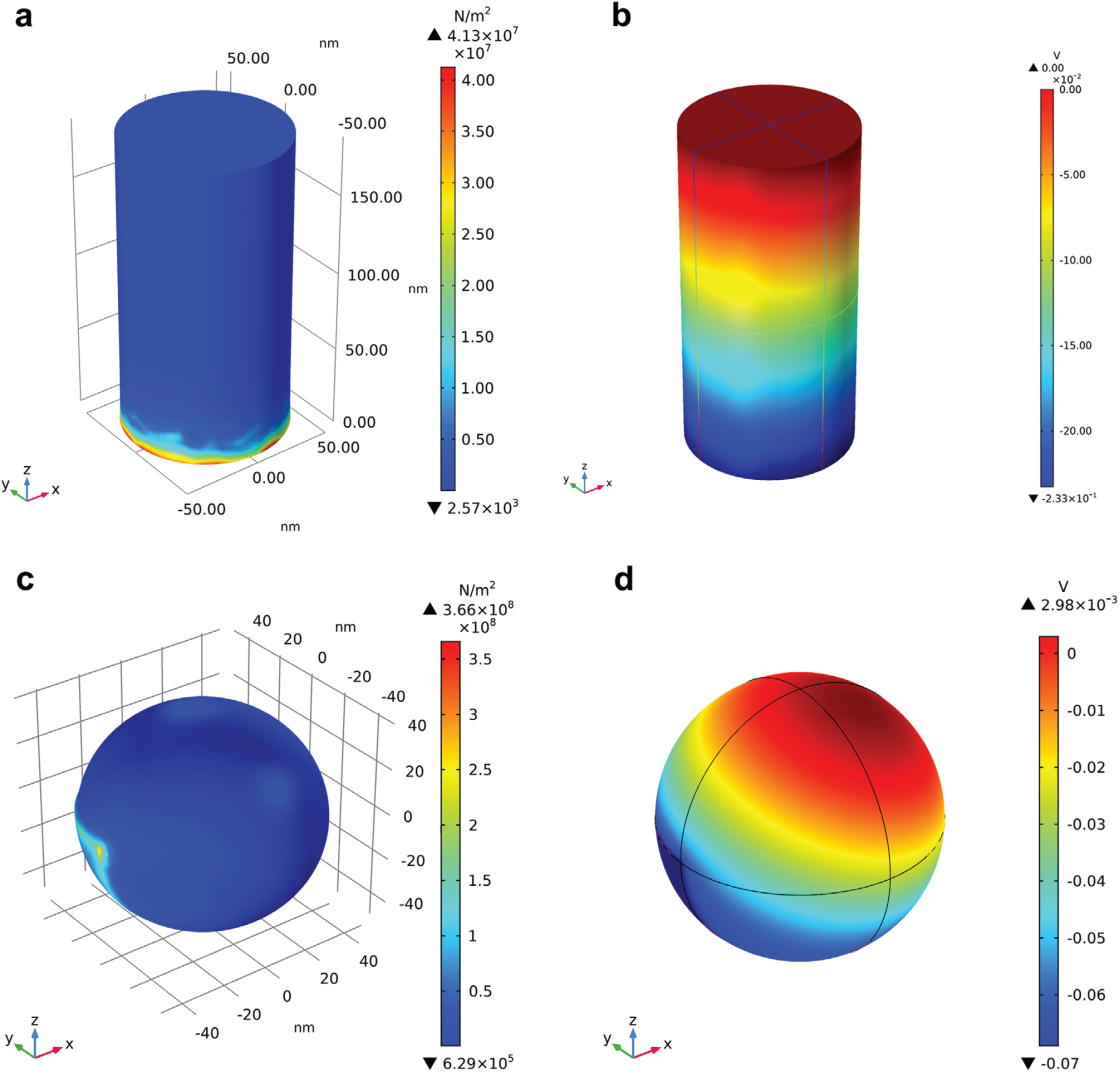
Finite element method simulation for the strain and piezopotential distribution on the surface of BaTiO_3_ a,b) nanorod and c,d) nanoparticle with the cavitation pressure of 10^8^ Pa.

Finite element analysis was used to theoretically calculate the surface piezopotential distribution on BaTiO_3_ under compressive strain. The geometries for the simulated models of BaTiO_3_ were derived from the electron microscopy images in Figure 2a and Figure S8, Supporting Information. The predicted piezopotential of the highly asymmetric BaTiO_3_ nanorods (0.23 V) with a length of 200 nm was much higher than that of BaTiO_3_ nanospheres (0.07 V) (**Figure** **5**). An identical mechanical strain was presumed. As expected, the piezopotential of the BaTiO_3_ nanorods decreased with a decreasing length or axial ratio. The BaTiO_3_ nanorod with a length of 120 nm possessed a predicted piezopotential of 0.18 V, subjected to a total strain of 10^8^ Pa (Figure S17, Supporting Information). Although the dielectric coefficient of tetragonal BaTiO_3_:Nb is different from that of BaTiO_3_, it was assumed that the overall geometric relationships between the particle anisotropy and the piezopotential would hold for BaTiO_3_:Nb. It can be concluded that the highly anisotropic nanoparticles in BaTiO_3_:Nb/C would generate a much higher piezopotential than the sphere‐like BaTiO_3_:Nb‐C. In other words, the high piezo‐ and piezophoto‐catalytic activities of BaTiO_3_:Nb/C can be ascribed to its nanorod‐like and highly anisotropic nature.

**Figure 6:**
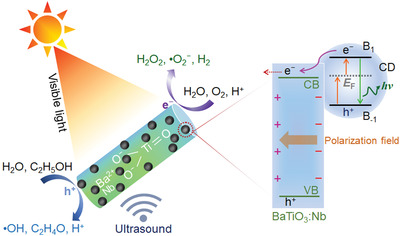
An illustration of the approach for catalytic production of H_2_O_2_ during simultaneous exposure to visible light and ultrasound with a catalyst based on BaTiO_3_:Nb and carbon quantum dots (CDs). Light irradiation of CDs results in the transfer of photoinduced electrons to the conduction band (CB) of BaTiO_3_:Nb, which in turn are involved in the reduction of O_2_. The electron donor C_2_H_5_OH quenches photoinduced holes (in the CDs and valance band [VB] of BaTiO_3_:Nb) and inhibits recombination of photoinduced charge carriers.

The working principle of piezophotocatalysis for the H_2_O_2_ generation with BaTiO_3_:Nb modified by CDs is illustrated in **Figure** **6**. When BaTiO_3_:Nb/C is irradiated with visible light, photons are absorbed by the CDs, and photoinduced electrons from the CDs are injected into the conduction band of BaTiO_3_:Nb. The holes left in the CDs oxidize H_2_O to •OH radicals (Figure S18a, Supporting Information) and ethanol to acetaldehyde (C_2_H_4_O).^[^ ^15^ ^]^ Due to the metallic character of CDs, photon absorption causes interband transitions either from a fully occupied band (B_−1_) to a partially occupied conduction band near to the Fermi level (*E*_F_) or, alternatively, from a conduction band to the lowest unoccupied band (B_1_), as shown on the right‐hand side of Figure 6. H^+^ will diffuse and adhere to the surface of BaTiO_3_:Nb, where they are reduced to H_2_ with electrons from the conduction band. Some O_2_ is also reduced by these electrons to •O_2_^−^ radicals (Figure S18b, Supporting Information). However, note that the sole photocatalytic activity of BaTiO_3_:Nb/C for the generation of H_2_O_2_ is comparably low. It might be ascribed to the recombination of photoinduced charge carriers at the interfaces of BaTiO_3_:Nb and CDs, and the low carrier density and electron mobility of BaTiO_3_:Nb.^[^ ^57^ ^]^ The partially photoinduced electrons in the CDs will also inevitably recombine with the holes and offset the excess energy in the form of scattered photons and heat. When introducing a polarization field via piezotronic effects, the utilization ratio of the photoinduced electrons is significantly improved. The induced electric polarization field in BaTiO_3_:Nb drives the photoinduced electrons to move toward specific crystalline planes, thus prohibiting the recombination of photoinduced electron–hole pairs. The photoinduced holes in the valance band of BaTiO_3_:Nb migrate in the opposite direction to the photoinduced electrons when the piezoelectric polarization field is applied. The oscillatory piezoelectric polarization field inhibits recombination of interface charges, which is beneficial to piezophotocatalysis. The CDs enhance the conductivity of the composite nanoparticles, which benefits mobility of the additional screening charges at the polarized BaTiO_3_:Nb nanoparticles and their interface with the ethanol‐water solvent. This screening leads to a higher catalytic generation of H_2_O_2_ compared to catalysts without CDs. Notably, the sole effect of piezocatalysis was also high although not as high as that for piezophotocatalysis.

## 3 Conclusions

In summary, we showed that a catalyst based on Nb‐doped BaTiO_3_ (BaTiO_3_:Nb) sensitized by CDs worked well for the generation of H_2_O_2_ from O_2_ and H_2_O in an ethanol–water solvent under ultrasound and visible light co‐irradiation. The rates of H_2_O_2_ generation with BaTiO_3_:Nb/C reached 1360 µmol g^−1^ h^−1^ during piezophotocatalysis, which was higher than for sole piezo‐ and photo‐catalysis. However, the generation rates were also high for sole piezocatalysis but much smaller for photocatalysis. The high performances were ascribed to the piezoelectric polarization field mediated by BaTiO_3_:Nb, in which charge carriers were injected from the CDs to the conduction band of BaTiO_3_:Nb. These could migrate to the surface of the catalyst particle without recombination, and then induce the involved redox reactions at the solid–liquid interface. This attribution was supported by the fact that the BaTiO_3_:Nb‐C with spherical structures had substantially smaller piezo(photo)catalytic activity than the BaTiO_3_:Nb/C with highly anisotropic geometries (nanorod structures). Equation‐driven calculations showed that an anisotropic geometry led to a high piezopotential. It was also shown that the deposition of CDs enhanced the conductivity of the catalysts, which was beneficial to the migration of surface‐screening charges induced by polarized charges, thus improving the piezocatalytic activities. The strategy that we used in this study shows that there is a way to use defects in semiconductors for the synthesis of H_2_O_2_ during piezo‐ and piezophoto‐catalysis. It may provide a new way for large‐scale synthesis of H_2_O_2_ using visible light and mechanical energy with the potential to replace current processes that use the oxidation of anthraquinone.

## 4 Experimental Section

### Synthesis of Nb‐doped BaTiO_3_ and Undoped BaTiO_3_

BaTiO_3_ doped with Nb was synthesized by a hydrothermal and post‐annealing method. Briefly, 1.0 g of PEG (its relative molecular weight was 6000) (Sinopharm, GR) was dissolved in 12 mL of a mixed solvent (volume ratio water:ethanol was 1:5) under stirring for 5 min to form a clear solution in flask A. 2.0 g of KOH (Sinopharm, ≥85.0%) was dissolved in 12 mL of a mixed solvent (volume ratio water:ethanol was 1:1) at room temperature under ultrasound irradiation for 3 min to form a clear solution in flask B. Then 340 µL of C_16_H_36_O_4_Ti (Sinopharm, 98.0%) and 2.7 mg of NbCl_5_ (Sinopharm, 99.9%) were dissolved in 10 mL of absolute ethanol and were dropwise added into flask A. The mixed content was stirred for another 30 min at room temperature. The contents of flask B were added into flask A and stirred for another 30 min at room temperature. Next, 0.3 g of BaCl 2H_2_O (Sinopharm, >99.0%) was added to the above reaction solution and stirred for 10 min. The white colloidal suspension was transferred to a Teflon‐lined stainless‐steel autoclave and put in an oven, where the heating rate was 5 °C min^−1^ and maintained at 200 °C for 12 h. The obtained precipitation was collected by centrifugation and washed three times with 0.5 mm of an aqueous solution of formic acid (Sinopharm, >98.0%) and deionized water, and dried at 100 °C for 12 h. The product was subsequently annealed in an alumina porcelain boat at 800 °C for 2 h in air and then allowed to cool naturally to room temperature. The theoretical Nb:Ba molar ratio of the BaTiO_3_:Nb prepared with this step was 1:100. For comparison, different BaTiO_3_:Nb were prepared by the same method with theoretical Nb:Ba molar ratios of 1:120 and 1:80. In addition, another kind of Nb‐doped BaTiO_3_ was synthesized by the same reaction steps; the only difference was to replace 1.0 g PEG with 0.1 g sodium oleate (C_18_H_33_NaO_2_; Sinopharm, >97.0%). The undoped BaTiO_3_ was synthesized by removing the addition of NbCl_5_ in the process of BaTiO_3_:Nb preparation.

### Carbon Quantum Dots Loading

The deposition of CDs was performed in situ by first dispersing 0.05 g BaTiO_3_:Nb in 60 mL of aqueous glucose (Sinopharm, AR) solution under ultrasound for 10 min to form a milky suspension. The concentration of glucose was 0.8 mm. Then the milky suspension was transferred to a Teflon‐lined stainless‐steel autoclave and placed in an oven, where the heating rate was 5 °C min^−1^ and maintained at 160 °C for 5 h. The amount of CDs loaded on the BaTiO_3_:Nb via this process should be slightly lower than the theoretically calculated 7.2 wt% CDs on BaTiO_3_:Nb/C. After the reactor was cooled naturally to room temperature, the obtained grey product was thoroughly washed with absolute ethanol three times and finally dried at 70 °C for 12 h in a dynamic vacuum. The catalyst with CDs that was synthesized with PEG as an additive was labeled as BaTiO_3_:Nb/C, and the one with CDs synthesized with C_18_H_33_NaO_2_ as an additive was labeled as BaTiO_3_:Nb‐C. To vary the CDs content loaded on the BaTiO_3_:Nb, additional aqueous solutions of glucose with 0.4, 1.2, and 1.6 mm were prepared and used as described above. Pure carbon was prepared as described above without adding BaTiO_3_:Nb. For comparison, the BaTiO_3_ modified with CDs was prepared with the same procedure.

### Characterization

Field‐emission SEM images of the samples were recorded with a Nova NanoSEM 450 (FEI, USA) instrument. Field‐emission TEM images, EDS, and SAED patterns were recorded on a Tecnai G2 F20 S‐Twin (FEL, USA). XRD patterns of the samples were recorded with a D/max2550VB3+/PC diffractometer (Rigaku, Japan) using a Cu K*α* source operated at 40 kV and 100 mA. Raman spectra of the samples were recorded on a LabRAM HR Evolution (Horiba, France) spectrometer with laser radiation of 532 nm. All Raman spectra were recorded at 25 °C under air. XPS spectra were acquired on an Escalab 250Xi (Thermo Scientific) device with an excitation source of Al K*α* radiation. All the binding energies of the Ba, O, Ti, and Nb elements were calibrated using the binding energy of C 1s. PFM was performed with a Dimension Icon atomic force microscope (Bruker, USA). Steady‐state PL spectra were recorded with an Edinburgh Analytical Instrument PLS980 coupled with a time‐correlated single‐photon‐counting system at room temperature, using a 4.0 eV excitation laser with 524 nm emission light. UV–vis diffuse reflectance spectra were recorded with a UV–vis‐near‐infrared spectrometer (U‐4100, Hitachi) equipped with an integrating sphere and were converted from reflectance into absorbance using the Kubelka–Munk function. Dynamic light scattering (DLS) measurements were conducted with a Malvern Zetasizer Nano ZS90 (FPMRC‐PA‐200I) at room temperature. EPR spectra were measured on a Bruker EMX‐10/12 spectrometer at room temperature. Finite element method calculations were carried out with COMSOL Multiphysics 5.4 with a model of a piezoelectric device based on a steady‐state study.

### Electrochemical Measurements

Electrochemical measurements were performed in 0.1 ^3^mol/dm Na_2_SO_4_ (pH = 6.8) as an electrolyte with an Ag/AgCl reference electrode (saturated aqueous solution of KCl) and a Pt wire counter‐electrode on a CHI 760E electrochemical workstation (Chenhua, China). The working electrodes were fabricated by a particle transfer method. Briefly, 50 mg of sample powders were suspended in 1.0 mL ethanol under ultrasound irradiation for 30 min and spin‐coated onto a fluorine tin oxide glass substrate (the effective area of the thin film was controlled at 1 × 1 cm^2^). After drying at room temperature, the electrode was heated to 80 °C for 12 h in a vacuum enhancing the adhesion between the sample and the glass. The impedance spectra were recorded in the frequency range of 10^5^–10 Hz unless otherwise noted, with an amplitude of 10 mV under darkness. Mott–Schottky curves were registered at frequencies of 500, 1000, and 1500 Hz. Electrochemical potentials were all expressed against RHE by *E*_RHE_ = *E*_Ag/AgCl_ + 0.059 pH + 0.1976 V. Photocurrent response curves of the samples were tested under the alternating illumination of visible light (on and off).

### Catalytic H_2_O_2_ Production

15 mg of as‐prepared catalysts were added into 30 mL of ethanol–water solution (the volume ratio ethanol:water was 1:9) in a 200 mL glass bottle (Φ, 64 mm) and stirred for 10 min in the dark to disperse the catalysts. Subsequently, the catalytic reactions were carried out under visible light irradiation (at *λ* > 420 nm and light intensity of 100 mW cm^−2^ with a cold light source of 300 W Xe lamp), ultrasound activation (at 40 kHz, 150 W), and visible light together with ultrasound irradiation to study photo‐, piezo‐, and piezophoto‐catalysis for H_2_O_2_ production, respectively. Setting the light wavelength to greater than 420 nm was mainly because the visible light in the solar spectrum exceeds 42%, and the higher the utilization of visible light by photocatalysts, the more conducive it was to its practical application. The choice of ultrasonic frequency and power was mainly that the frequency and power of commercial ultrasonic cleaners were mostly set in this range. The ultrasonic wave was created by a digital ultrasonic cleaner (Skymen JP‐060s, 330 × 300 × 150 mm^3^) during piezo‐ and piezophoto‐catalysis in the present reaction system, and the water level in the ultrasonic cleaner was ≈6 cm to ensure that the glass bottle floats on the water surface. The temperature of the reaction solution was controlled by a long‐running water bath in the studies of the piezo‐ and piezophoto‐catalysis. The distance between the light source and the surface of the reaction solution was fixed at 10 cm. The produced concentration of H_2_O_2_ was determined by redox titration with KI (Sinopharm, >99.0%) aqueous solution, according to the authors' reported procedures.^[^ ^58^ ^]^ For the action spectrum analysis, the reactions were performed at 298 K under monochromated light irradiation, with *Φ*_AQY_ determined by Equation (3)^[^ ^59^ ^]^

(3) $$\Phi_{\mathrm{AQY}}\left(\%\right)=\frac{2\;\times N(H_{2}O_{2})}{N\left(\mathrm{Photons}\right)}\times 100$$

where *N*(H_2_O_2_) and *N*(Photons) represented the number of H_2_O_2_ molecules generated and the number of photons reaching the surface of the reaction solution.

The SCC efficiency was measured under simulated sunlight irradiation (AM1.5G, solar simulator XHA, China). The SCC efficiency was determined according to the following Equation (4)^[^ ^58b^ ^]^

(4) $$\begin{array}{ccc} & & \text{SCC}\;\text{efficiency}\;\left(\%\right)=\\ & & \frac{\left[\Delta G\;\text{for}\;H_{2}O_{2}\;\text{generation}\;\left(J\;\text{mo}l^{-1}\right)\right]\times \;\left[H_{2}O_{2}\;\text{formed}\;\left(\mathrm{mol}\right)\right]}{\left[\text{total}\;\text{input}\;\text{energy}\;\left(W\right)\right]\times \;\left[\text{reaction}\;\text{time}\;\left(s\right)\right]}\\ & & \times 100 \end{array}$$

The free energy for H_2_O_2_ generation was 117 kJ mol^−1^, the irradiance of the AM1.5 global spectrum equipped with a cutoff filter (*λ* > 420 nm) was 100 mW cm^−2^, and the irradiated area was 3.14 × 10^−4^ m^2^. The total input energy was 0.314 W. After the reactions, the catalysts were recovered by centrifugation, and the produced amount of H_2_O_2_ in the solution was quantified by the iodometric titration.

### Photocatalytic Reactions for Overall Water Splitting

The catalysts (15 mg) and ethanol–water solution (30 mL, the volume ratio ethanol:water was 1:9) were added to an overhead‐irradiation‐type glass vessel connected to a closed gas circulation system, and the distance between the light source and solution surface was fixed at 10 cm. Before each reaction, all air was evacuated from the reaction system and filled with Ar (about 1 kPa unless otherwise noted). The suspension was subsequently irradiated using a 300 W Xe lamp (PLS‐SXE300D, 100 mW cm^−2^, full arc) with a 420 nm cutoff filter as the visible light source. Evolved gases accumulated in the closed gas circulation system were analyzed by gas chromatography (GC9790II, Ar carrier gas).

### Statistical Analysis

Statistical analyses for the amount of H_2_O_2_ formed were carried out using the calibration curve between the absorbance of a solution containing I_3_^−^ ions and concentration of H_2_O_2_, as shown in Figure S19, Supporting Information. The error bar represented a typical error as determined from three separate experiments, which was the difference between the maximum and minimum values of the data from three tests. Results were presented as arithmetic means from three tests and with a corresponding error bar. Preprocessing of the data in relation to the H_2_O_2_ concentration measurements, the volume of H_2_ produced, (photo)electrochemical curves (such as Mott–Schottky curves, transient photocurrent curves, and EIS plots), PL spectra, and particles size distribution derived from DLS method were not performed; no outliers were removed. The intensity for the X‐ray diffractograms, Raman spectra, and diffuse reflection spectra are presented in Figure 3b–d on arbitrary intensity scales and the individual traces were not visualized on the same absolute intensity scales. Also, the information in Figures S4, S7, S8, and S18, Supporting Information, were presented on arbitrary intensity scales where the absolute intensities were not the same.

## Conflict of Interest

The authors declare no conflict of interest.

## Supporting information

Supporting Information

## Data Availability

The data that support the findings of this study are available from the corresponding author upon reasonable request.

## References

[1] a) S. C. Perry , D. Pangotra , L. Vieira , L.‐I. Csepei , V. Sieber , L. Wang , C. P. de León , F. C. Walsh , Nat. Rev. Chem. 2019, 3, 442;

[2] K. Oka , H. Nishide , B. Winther‐Jensen , Adv. Sci. 2021, 8, 2003077. 10.1002/advs.202003077 PMC7927612 33717849

[3] S. Fukuzumi , Joule 2017, 1, 689.

[4] J. M. Campos‐Martin , G. Blanco‐Brieva , J. L. G. Fierro , Angew. Chem., Int. Ed. 2006, 45, 6962. 10.1002/anie.200503779 17039551

[5] J. García‐Serna , T. Moreno , P. Biasi , M. J. Cocero , J.‐P. Mikkola , T. O. Salmi , Green Chem. 2014, 16, 2320.

[6] a) C. Krishnaraj , H. S. Jena , L. Bourda , A. Laemont , P. Pachfule , J. Roeser , C. V. Chandran , S. Borgmans , S. M. J. Rogge , K. Leus , C. V. Stevens , J. A. Martens , V. Van Speybroeck , E. Breynaert , A. Thomas , P. Van Der Voort , J. Am. Chem. Soc. 2020, 142, 20107; 33185433 10.1021/jacs.0c09684 PMC7705891

[7] a) Z. Teng , Q. Zhang , H. Yang , K. Kato , W. Yang , Y.‐R. Lu , S. Liu , C. Wang , A. Yamakata , C. Su , B. Liu , T. Ohno , Nat. Catal. 2021, 4, 374;

[8] D. Tsukamoto , A. Shiro , Y. Shiraishi , Y. Sugano , S. Ichikawa , S. Tanaka , T. Hirai , ACS Catal. 2012, 2, 599.

[9] H. Park , W. Choi , J. Phys. Chem. B 2004, 108, 4086. 10.1021/jp049789g 18950128

[10] N. O. Balayeva , N. Zheng , R. Dillert , D. W. Bahnemann , ACS Catal. 2019, 9, 10694.

[11] O. Jung , M. L. Pegis , Z. Wang , G. Banerjee , C. T. Nemes , W. L. Hoffeditz , J. T. Hupp , C. A. Schmuttenmaer , G. W. Brudvig , J. M. Mayer , J. Am. Chem. Soc. 2018, 140, 4079. 29463086 10.1021/jacs.8b00015

[12] C. Hu , F. Chen , Y. Wang , N. Tian , T. Ma , Y. Zhang , H. Huang , Adv. Mater. 2021, 33, 2101751. 10.1002/adma.202101751 33963776

[13] a) J. Yoon , J. Kim , F. Tieves , W. Zhang , M. Alcalde , F. Hollmann , C. B. Park , ACS Catal. 2020, 10, 5236;

[14] a) E. Lin , N. Qin , J. Wu , B. Yuan , Z. Kang , D. Bao , ACS Appl. Mater. Interfaces 2020, 12, 14005; 32142247 10.1021/acsami.0c00962

[15] X. Zhou , B. Shen , J. Zhai , J. C. Conesa , Small Methods 2021, 5, 2100269. 10.1002/smtd.202100269 34927907

[16] T. D. Raju , S. Veeralingam , S. Badhulika , ACS Appl. Nano Mater. 2020, 3, 4777.

[17] a) H. You , Z. Wu , L. Zhang , Y. Ying , Y. Liu , L. Fei , X. Chen , Y. Jia , Y. Wang , F. Wang , S. Ju , J. Qiao , C.‐H. Lam , H. Huang , Angew. Chem., Int. Ed. 2019, 58, 11779; 10.1002/anie.201906181 31225687

[18] Y.‐C. Wang , J. M. Wu , Adv. Funct. Mater. 2020, 30, 1907619.

[19] M. B. Starr , J. Shi , X. Wang , Angew. Chem., Int. Ed. 2012, 51, 5962. 10.1002/anie.201201424 22556008

[20] Y.‐T. Lin , S.‐N. Lai , J. M. Wu , Adv. Mater. 2020, 32, 2002875.

[21] M. Laurenti , N. Garino , G. Canavese , S. Hernandéz , V. Cauda , ACS Appl. Mater. Interfaces 2020, 12, 25798. 32396322 10.1021/acsami.0c03787

[22] X. Zhou , F. Yan , S. Wu , B. Shen , H. Zeng , J. Zhai , Small 2020, 16, 2001573. 10.1002/smll.202001573 32431007

[23] F. Li , L. Cheng , J. Fan , Q. Xiang , J. Mater. Chem. A 2021, 9, 23765.

[24] Y. Li , X. Li , H. Zhang , J. Fan , Q. Xiang , J. Mater. Sci. Technol. 2020, 56, 69.

[25] a) L. Pan , S. Sun , Y. Chen , P. Wang , J. Wang , X. Zhang , J.‐J. Zou , Z. L. Wang , Adv. Energy Mater. 2020, 10, 2000214;

[26] a) L. Wang , S. Liu , Z. Wang , Y. Zhou , Y. Qin , Z. L. Wang , ACS Nano 2016, 10, 2636; 26745209 10.1021/acsnano.5b07678

[27] C. Hu , H. Huang , F. Chen , Y. Zhang , H. Yu , T. Ma , Adv. Funct. Mater. 2020, 30, 1908168.

[28] R. Su , Z. Wang , L. Zhu , Y. Pan , D. Zhang , H. Wen , Z.‐D. Luo , L. Li , F.‐t. Li , M. Wu , L. He , P. Sharma , J. Seidel , Angew. Chem., Int. Ed. 2021, 60, 16019. 10.1002/anie.202103112 33871146

[29] N. A. Benedek , C. J. Fennie , J. Phys. Chem. C 2013, 117, 13339.

[30] a) B. Jiang , J. Iocozzia , L. Zhao , H. Zhang , Y.‐W. Harn , Y. Chen , Z. Lin , Chem. Soc. Rev. 2019, 48, 1194; 30663742 10.1039/c8cs00583d

[31] R. Su , H. A. Hsain , M. Wu , D. Zhang , X. Hu , Z. Wang , X. Wang , F.‐t. Li , X. Chen , L. Zhu , Y. Yang , Y. Yang , X. Lou , S. J. Pennycook , Angew. Chem., Int. Ed. 2019, 58, 15076. 10.1002/anie.201907695 31404487

[32] S. Das , S. Ghara , P. Mahadevan , A. Sundaresan , J. Gopalakrishnan , D. D. Sarma , ACS Energy Lett. 2018, 3, 1176.

[33] N. Masó , H. Beltrán , E. Cordoncillo , A. A. Flores , P. Escribano , D. C. Sinclair , A. R. West , J. Mater. Chem. 2006, 16, 3114.

[34] B. C. M. Martindale , G. A. M. Hutton , C. A. Caputo , E. Reisner , J. Am. Chem. Soc. 2015, 137, 6018. 25864839 10.1021/jacs.5b01650

[35] a) J. Tuček , K. C. Kemp , K. S. Kim , R. Zbořil , ACS Nano 2014, 8, 7571; 25000534 10.1021/nn501836x

[36] W. Wu , L. Zhan , K. Ohkubo , Y. Yamada , M. Wu , S. Fukuzumi , J. Photochem. Photobiol., B 2015, 152, 63. 25498411 10.1016/j.jphotobiol.2014.10.018

[37] a) L. Cao , S. Sahu , P. Anilkumar , C. E. Bunker , J. Xu , K. A. S. Fernando , P. Wang , E. A. Guliants , K. N. Tackett , Y.‐P. Sun , J. Am. Chem. Soc. 2011, 133, 4754; 21401091 10.1021/ja200804h

[38] H. Peng , J. Travas‐Sejdic , Chem. Mater. 2009, 21, 5563.

[39] a) M.‐M. Titirici , R. J. White , N. Brun , V. L. Budarin , D. S. Su , F. del Monte , J. H. Clark , M. J. MacLachlan , Chem. Soc. Rev. 2015, 44, 250; 25301517 10.1039/c4cs00232f

[40] S. H. Jin , D. H. Kim , G. H. Jun , S. H. Hong , S. Jeon , ACS Nano 2013, 7, 1239. 23272894 10.1021/nn304675g

[41] a) C. Falco , N. Baccile , M.‐M. Titirici , Green Chem. 2011, 13, 3273;

[42] Y. Wang , L. Zhang , J. Wang , Q. Li , H. Wang , L. Gu , J. Chen , J. Deng , K. Lin , L. Huang , X. Xing , J. Am. Chem. Soc. 2021, 143, 6491. 33900066 10.1021/jacs.1c00605

[43] S. Kobayashi , Y. Mizumukai , T. Ohnishi , N. Shibata , Y. Ikuhara , T. Yamamoto , ACS Nano 2015, 9, 10769. 26487067 10.1021/acsnano.5b05720

[44] N. Baccile , G. Laurent , F. Babonneau , F. Fayon , M.‐M. Titirici , M. Antonietti , J. Phys. Chem. C 2009, 113, 9644.

[45] H. Zhang , B. Cheng , Q. Li , B. Liu , Y. Mao , J. Phys. Chem. C 2018, 122, 5188.

[46] a) X. Li , L. Liang , Y. Sun , J. Xu , X. Jiao , X. Xu , H. Ju , Y. Pan , J. Zhu , Y. Xie , J. Am. Chem. Soc. 2019, 141, 423; 30537829 10.1021/jacs.8b10692

[47] M. Kim , B. Lee , H. Ju , J. Y. Kim , J. Kim , S. W. Lee , Adv. Mater. 2019, 31, 1903316.

[48] J. Y. Y. Loh , Y. Ye , N. P. Kherani , ACS Appl. Mater. Interfaces 2020, 12, 2234. 31846296 10.1021/acsami.9b14097

[49] Y.‐X. Pan , Y. You , S. Xin , Y. Li , G. Fu , Z. Cui , Y.‐L. Men , F.‐F. Cao , S.‐H. Yu , J. B. Goodenough , J. Am. Chem. Soc. 2017, 139, 4123. 28215081 10.1021/jacs.7b00266

[50] H. Zhu , Y. Wang , J. Xiao , M. Liu , S. Xiong , Z. J. Wong , Z. Ye , Y. Ye , X. Yin , X. Zhang , Nat. Nanotechnol. 2015, 10, 151. 25531085 10.1038/nnano.2014.309

[51] M. Minary‐Jolandan , R. A. Bernal , I. Kuljanishvili , V. Parpoil , H. D. Espinosa , Nano Lett. 2012, 12, 970. 22191483 10.1021/nl204043y

[52] a) C. Dai , S. Zhang , Z. Liu , R. Wu , Y. Chen , ACS Nano 2017, 11, 9467; 28829584 10.1021/acsnano.7b05215

[53] J. Lv , T. Kako , Z. Li , Z. Zou , J. Ye , J. Phys. Chem. C 2010, 114, 6157.

[54] Y.‐X. Ye , J. Pan , F. Xie , L. Gong , S. Huang , Z. Ke , F. Zhu , J. Xu , G. Ouyang , Proc. Natl. Acad. Sci. U. S. A. 2021, 118, 2103964118. 10.1073/pnas.2103964118 PMC8072241 33853952

[55] Y. Zhang , L. Yan , M. Guan , D. Chen , Z. Xu , H. Guo , S. Hu , S. Zhang , X. Liu , Z. Guo , S. Li , S. Meng , Adv. Sci. 2022, 9, 2102978. 10.1002/advs.202102978 PMC8805563 34766740

[56] D. Yu , Z. Liu , J. Zhang , S. Li , Z. Zhao , L. Zhu , W. Liu , Y. Lin , H. Liu , Z. Zhang , Nano Energy 2019, 58, 695.

[57] K. Yasui , K. Kato , J. Phys. Chem. C 2013, 117, 19632.

[58] X. Zhou , B. Shen , J. Zhai , N. Hedin , Adv. Funct. Mater. 2021, 31, 2009594.

[59] T. Takata , J. Jiang , Y. Sakata , M. Nakabayashi , N. Shibata , V. Nandal , K. Seki , T. Hisatomi , K. Domen , Nature 2020, 581, 411. 32461647 10.1038/s41586-020-2278-9

