# Supplementary material for: Efficient Production of Solar Hydrogen Peroxide Using Piezoelectric Polarization and Photoinduced Charge Transfer of Nanopiezoelectrics Sensitized by Carbon Quantum Dots
Source: Adv Sci (Weinh). 2022 Apr 22;9(18):2105792. doi: 10.1002/advs.202105792 (PMC9218770; doi:10.1002/advs.202105792)
Supplement: Supplementary file 1 — Supporting Information [file ADVS-9-2105792-s001.pdf]

ADVANCED SCIENCE
Open Access

Supporting Information

for *Adv. Sci.*, DOI 10.1002/advs.202105792

Efficient Production of Solar Hydrogen Peroxide Using Piezoelectric Polarization and Photoinduced Charge Transfer of Nanopiezoelectrics Sensitized by Carbon Quantum Dots

Xiaofeng Zhou, Fei Yan, Alexander Lyubartsev, Bo Shen, Jiwei Zhai, José C. Conesa and Niklas Hedin**

Supporting Information

Efficient Production of Solar Hydrogen Peroxide using Piezoelectric Polarization and Photoinduced Charge Transfer of Nanopiezoelectrics Sensitized by Carbon Quantum Dots

Xiaofeng Zhou, Fei Yan, Alexander Lyubartsev, Bo Shen, Jiwei Zhai*, José C. Conesa, Niklas Hedin*

X. Zhou, F. Yan, Dr. B. Shen, Prof. J. Zhai
Shanghai Key Laboratory for R&D and Application of Metallic Functional Materials, Functional Materials Research Laboratory, School of Materials Science and Engineering, Tongji University, Shanghai 201804, China
E-mail: apzhai@tongji.edu.cn
X. Zhou, Prof. A. Lyubartsev, Prof. N. Hedin
Department of Materials and Environmental Chemistry, Stockholm University, Stockholm SE 106 91, Sweden
E-mail: niklas.hedin@mmk.su.se
Prof. J. C. Conesa
Instituto de Catálisis y Petroleoquímica, CSIC, Marie Curie 2, Cantoblanco, Madrid 28049, Spain

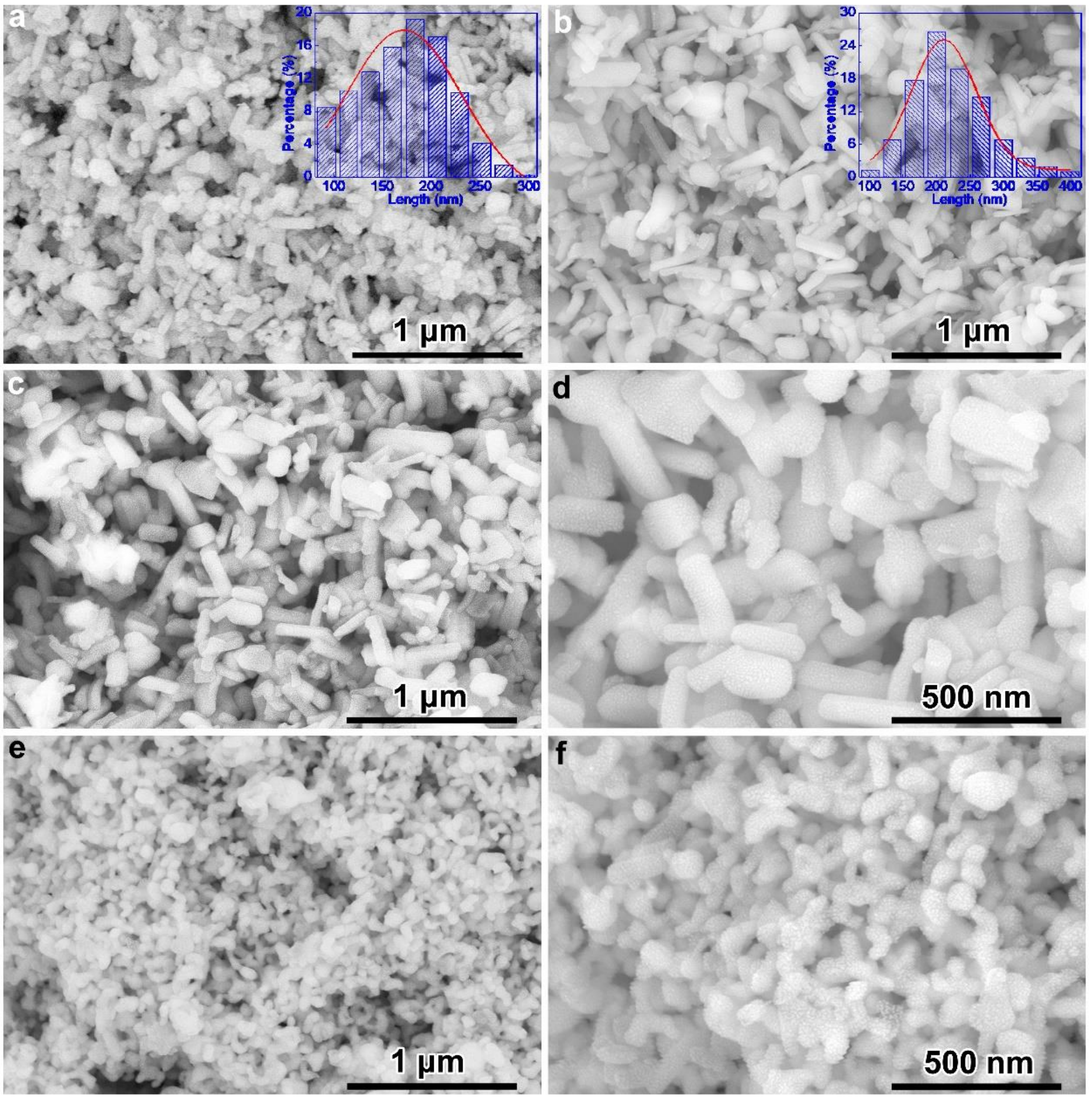

Figure S1. Scanning electron microscopic (SEM) images of pure a) $BaTiO_3$ and b) $BaTiO_3$:Nb prepared with poly(ethylene glycol) (PEG) addition, and the insets show the particle size distributions of $BaTiO_3$ and $BaTiO_3$:Nb; c,d) $BaTiO_3$:Nb/C prepared with PEG addition and e,f) $BaTiO_3$:Nb-C $BaTiO_3$:Nb prepared with sodium oleate addition.

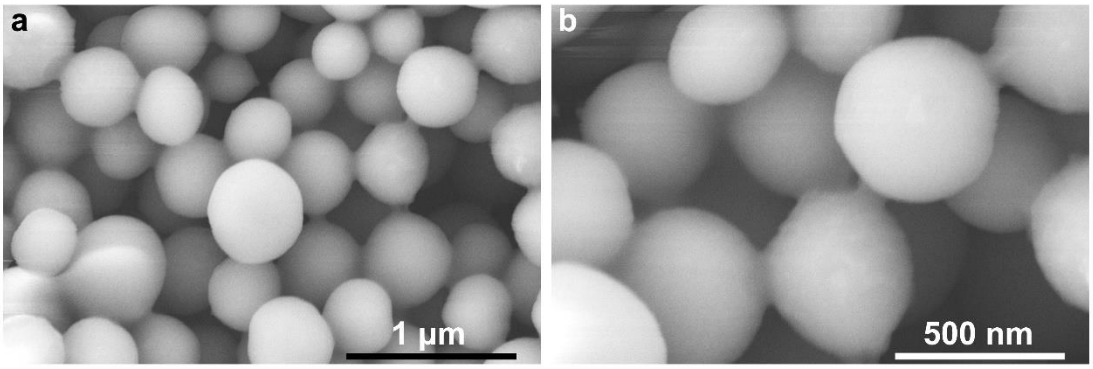

Figure S2. SEM images at lower a) and higher b) magnifications of hydrochar prepared by wet pyrolysis of an aqueous solution of glycose.

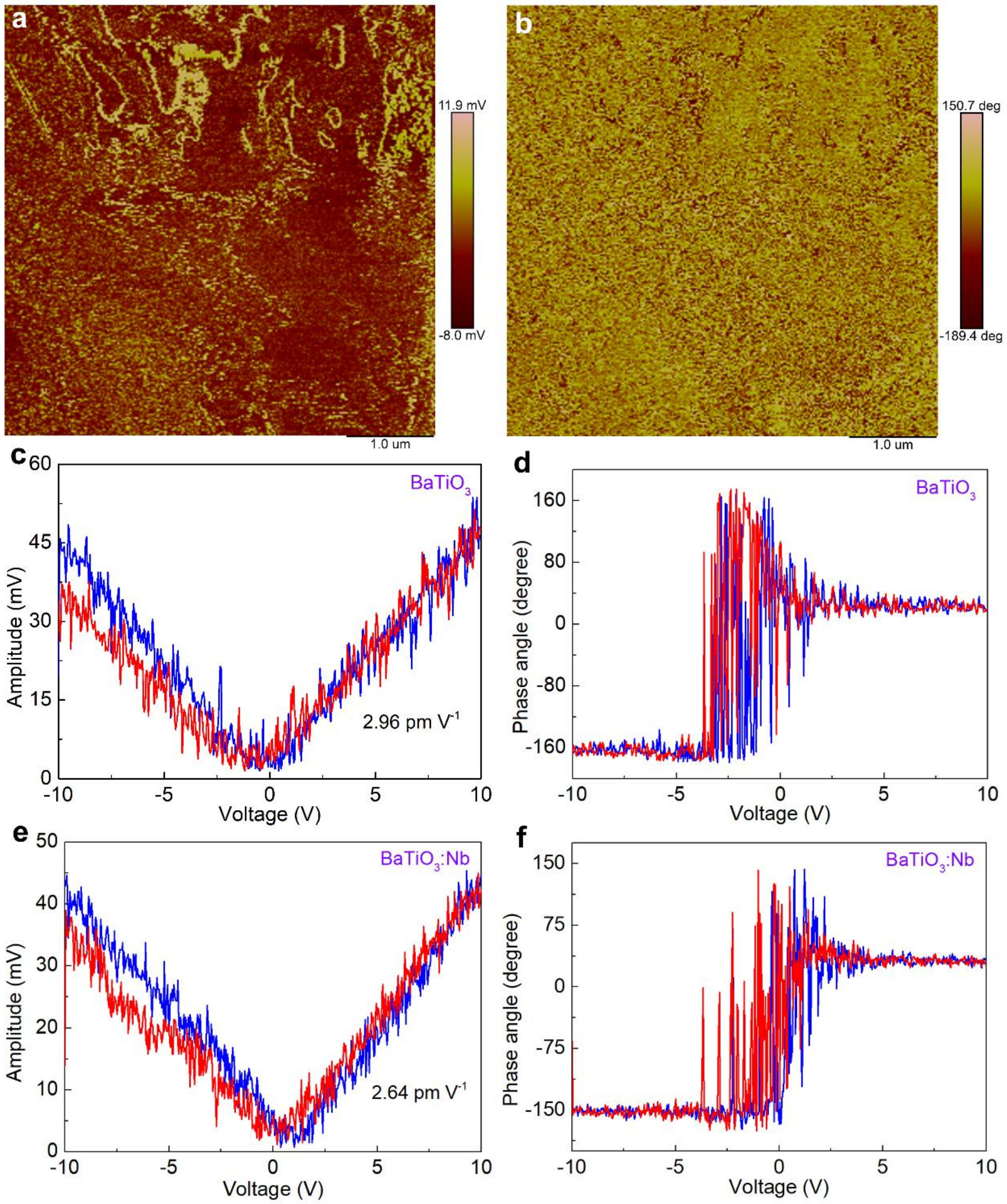

Figure S3. The out-of-plane piezoresponse force microscope images of $BaTiO_3$ for a) amplitude, b) phase, c) amplitude-voltage curve and d) phase-voltage curve. The out-of-plane piezoresponse force microscope images of $BaTiO_3$:Nb for e) amplitude-voltage curve and f) phase-voltage curve.

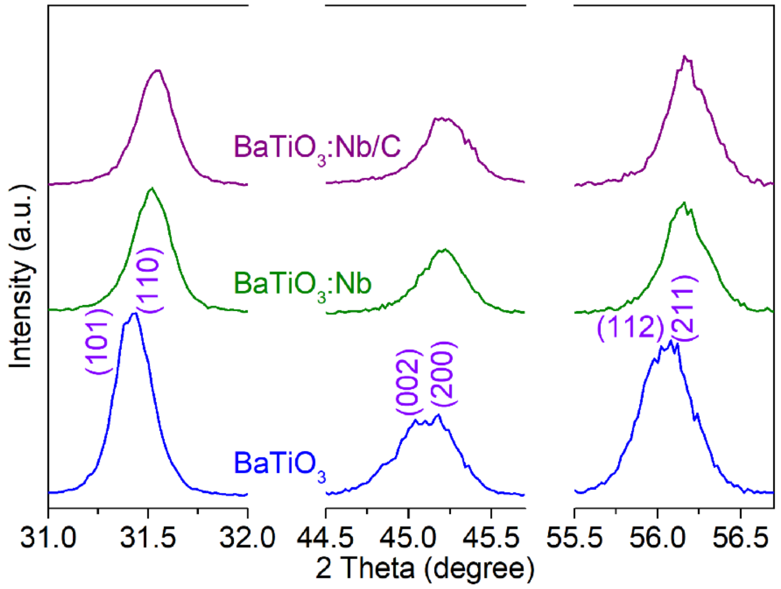

Figure S4. XRD local patterns of $BaTiO_3$, $BaTiO_3$:Nb and $BaTiO_3$:Nb/C.

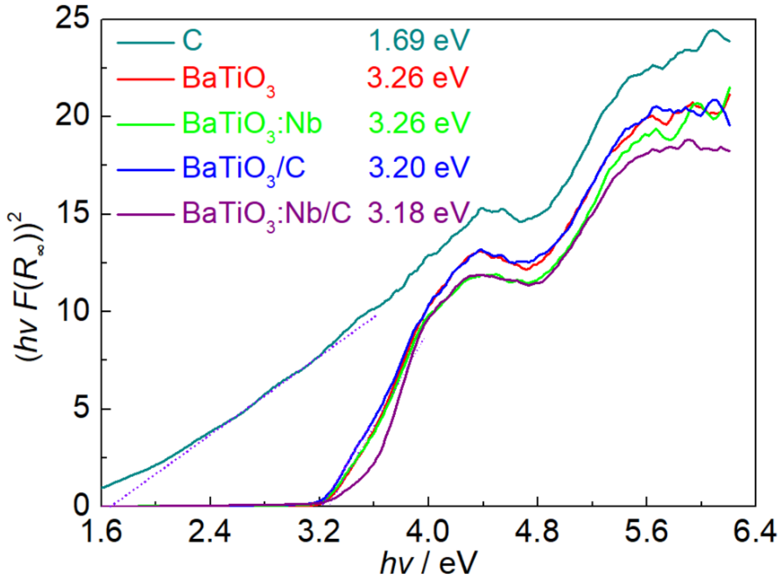

Figure S5. The bandgaps of different samples derived from diffused reflectance UV-Vis absorption spectra.

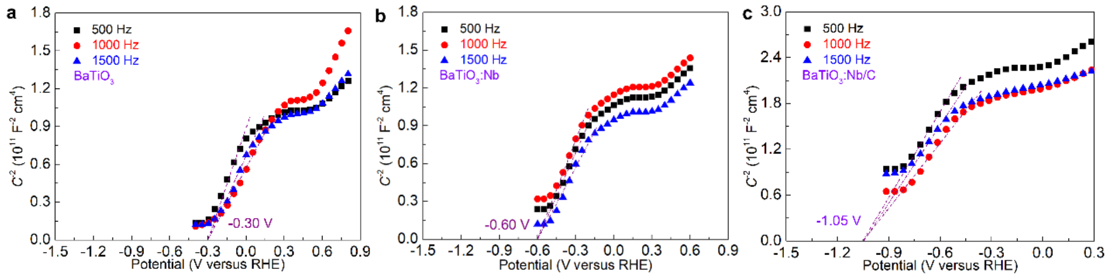

Figure S6. Electrochemical Motte-Schottky curves of $BaTiO_3$, $BaTiO_3$:Nb, and $BaTiO_3$:Nb/C recorded under test frequencies of 500, 1000, and 1500 Hz.

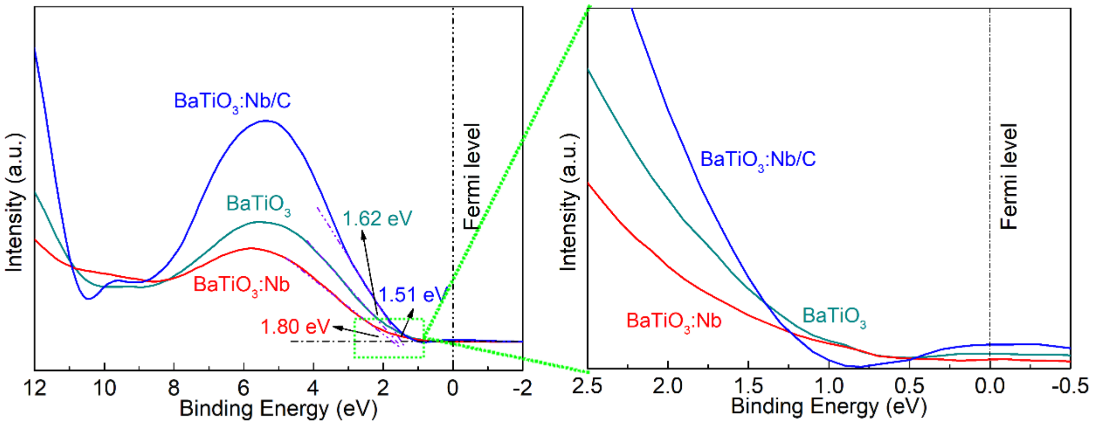

Figure S7. XPS valence spectra of $BaTiO_3$, $BaTiO_3$:Nb, and $BaTiO_3$:Nb/C. The right figure is a partial enlargement relative to the left figure.

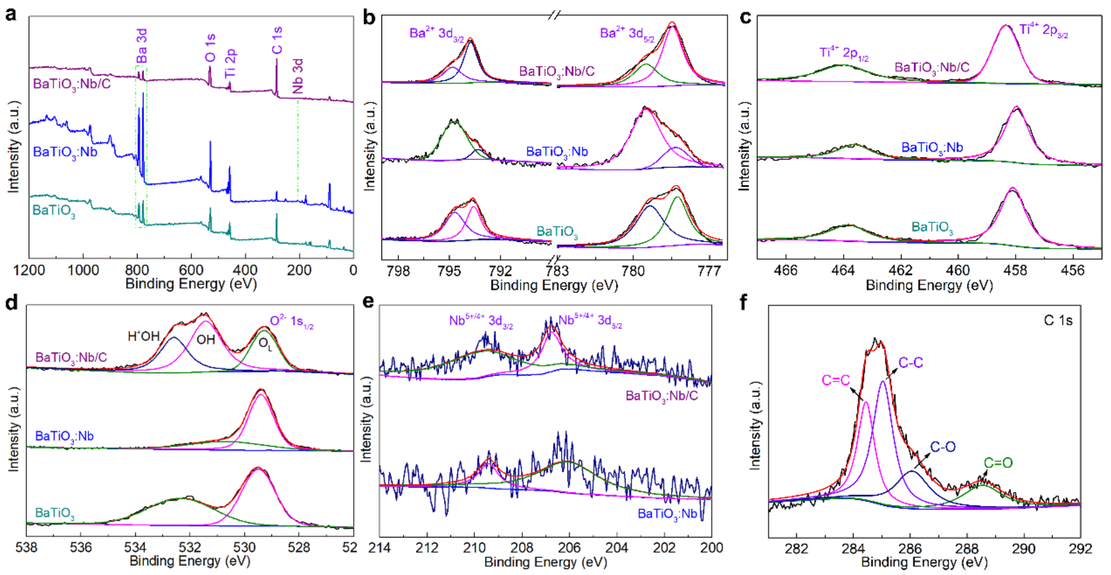

Figure S8. a) XPS survey spectra of $BaTiO_3$, $BaTiO_3$:Nb and $BaTiO_3$:Nb/C. XPS b) Ba 3d, c) Ti 2p and d) O 1s spectra of $BaTiO_3$, $BaTiO_3$:Nb and $BaTiO_3$:Nb/C. e) XPS Nd 3d spectra of $BaTiO_3$:Nb and $BaTiO_3$:Nb/C. f) XPS C 1s spectrum of $BaTiO_3$:Nb/C.

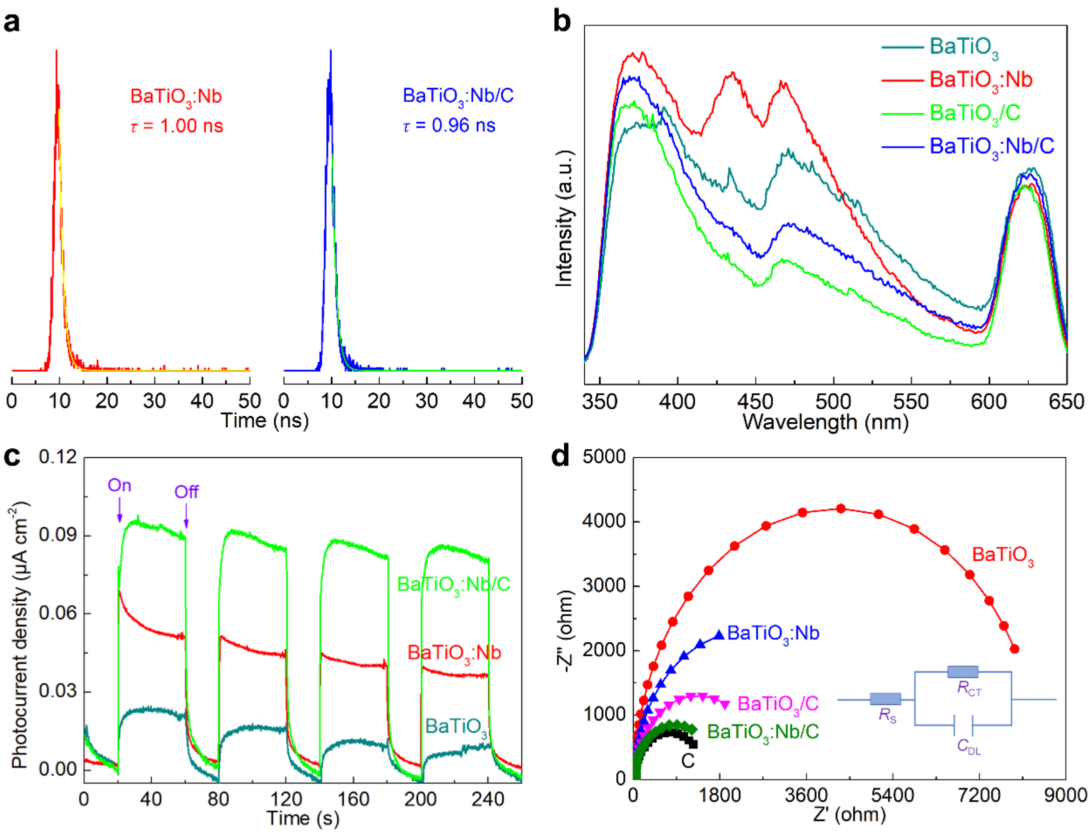

Figure S9. a) Time-resolved transient photoluminescence (PL) spectral decay of $BaTiO_3$:Nb and $BaTiO_3$:Nb/C. b) Steady-state PL spectra of $BaTiO_3$, $BaTiO_3$:Nb and $BaTiO_3$:Nb/C. c) Transient photocurrent curves of $BaTiO_3$, $BaTiO_3$:Nb and $BaTiO_3$:Nb/C measured in 0.1 M KCl under visible light irradiation. d) Electrochemical impedance spectroscopy (EIS) Nyquist plots of various catalysts measured in 0.1 M KCl in darkness. Inset, equivalent circuit model, comprising ohmic resistance (R_S), double-layer capacitance (C_{DL}) and charge transfer resistance (R_{CT}).

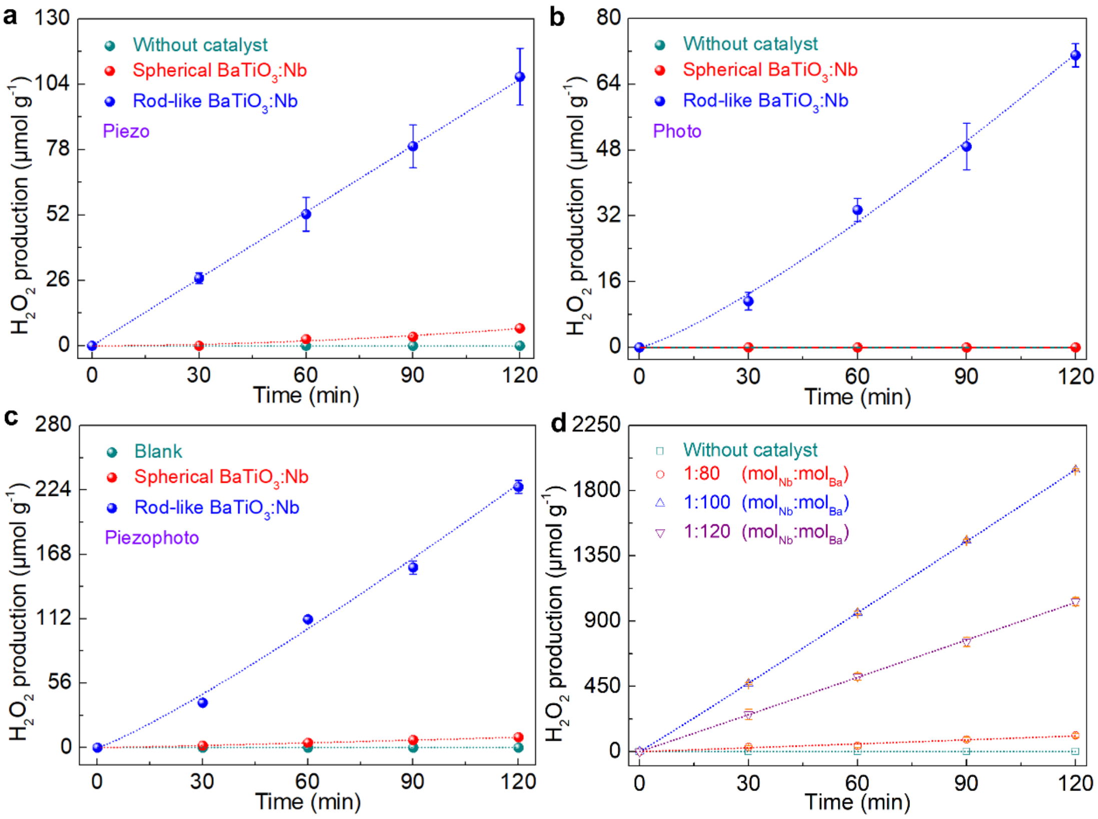

Figure S10. Time dependencies of the amount of H_2O_2 formed with rod-like or spherical $BaTiO_3$:Nb in an ethanol-water solvent under a) 40 kHz, 150 W ultrasonic vibration, b) visible light ($\lambda > 420$ nm) irradiation and c) ultrasound (40 kHz, 150 W) together with visible light ($\lambda > 420$ nm) irradiation. d) Time dependencies of the rate of H_2O_2 formed in an ethanol-water solvent with various Nb-doped $BaTiO_3$ under ultrasound (40 kHz, 150 W) together with visible light ($\lambda > 420$ nm) irradiation.

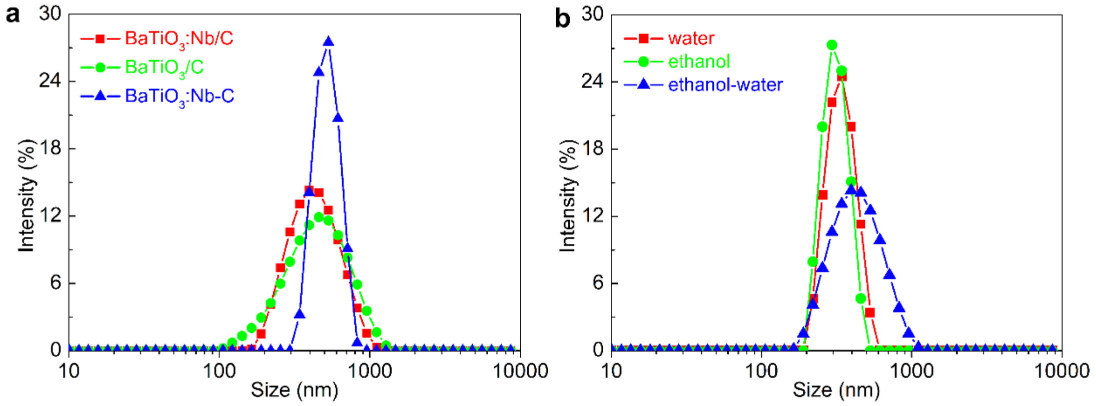

Figure S11. a) Particle size distributions of the $BaTiO_3$:Nb/C, $BaTiO_3$/C, and $BaTiO_3$:Nb-C in an ethanol-water solvent with dynamic light scattering method. b) Particle size distributions of the $BaTiO_3$:Nb/C in water, ethanol, and ethanol-water solvents with dynamic light scattering method.

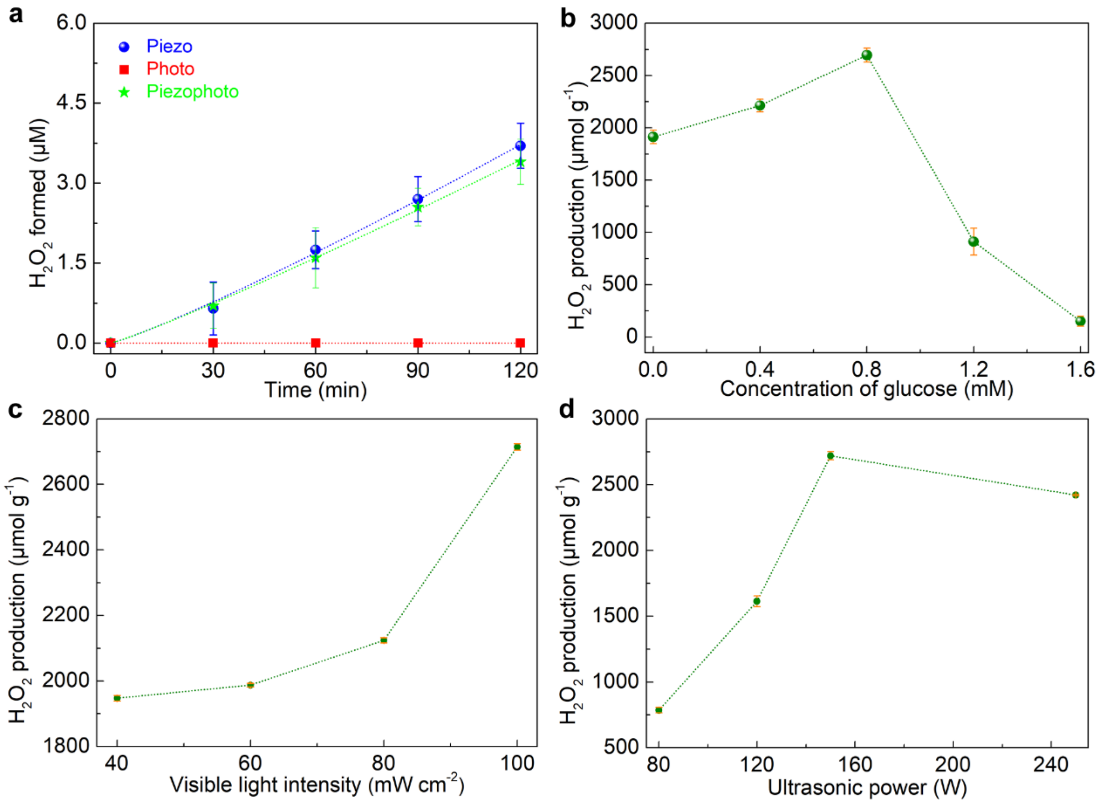

Figure S12. a) Time dependencies of the amount of H_2O_2 formed in an ethanol-water solvent without catalysts under a) 40 kHz, 150 W ultrasonic vibration (piezo), b) visible light (λ > 420 nm) irradiation (photo) and c) ultrasound (40 kHz, 150 W) together with visible light (λ > 420 nm) irradiation (piezophoto). b) Dependency of H_2O_2 production with rod-like $BaTiO_3$:Nb/C in an ethanol-water solvent under co-irradiation with ultrasound (40 kHz, 150 W) and visible light (λ > 420 nm, 100 mW cm^{-2}) for catalyst prepared with different concentrations of glucose during the wet pyrolysis processes. Dependencies of H_2O_2 production with rod-like $BaTiO_3$:Nb/C in an ethanol-water solvent under co-irradiation with ultrasound (40 kHz, 150 W) and visible light (λ > 420 nm, 100 mW cm^{-2}) on c) visible light intensity and d) ultrasonic power.

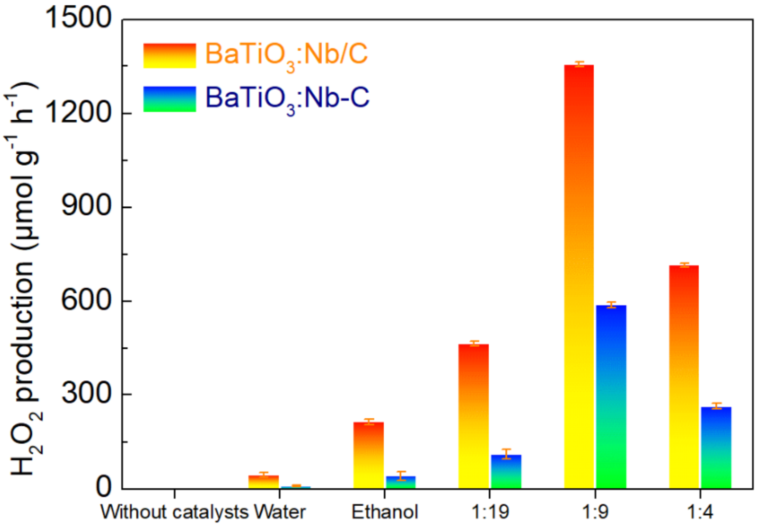

Figure S13. Comparison of H_2O_2 production rates for $BaTiO_3$:Nb/C and $BaTiO_3$:Nb-C in pure water, ethanol, or ethanol-water (the volume ratios of ethanol:water were 1:19, 1:9 and 1:4, respectively) solvent under ultrasound (40 kHz, 150 W) and visible light (λ > 420 nm, 100 mW cm^{-2}) co-irradiation.

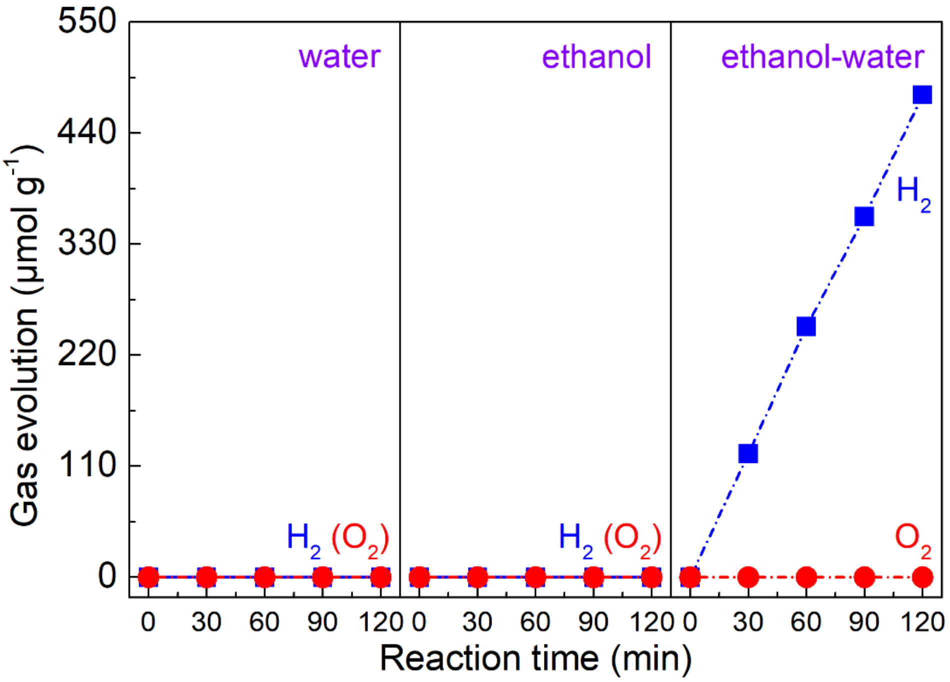

Figure S14. Time dependencies of water-splitting reaction on $BaTiO_3$:Nb/C in pure water, ethanol and ethanol-water solvents under visible light irradiation.

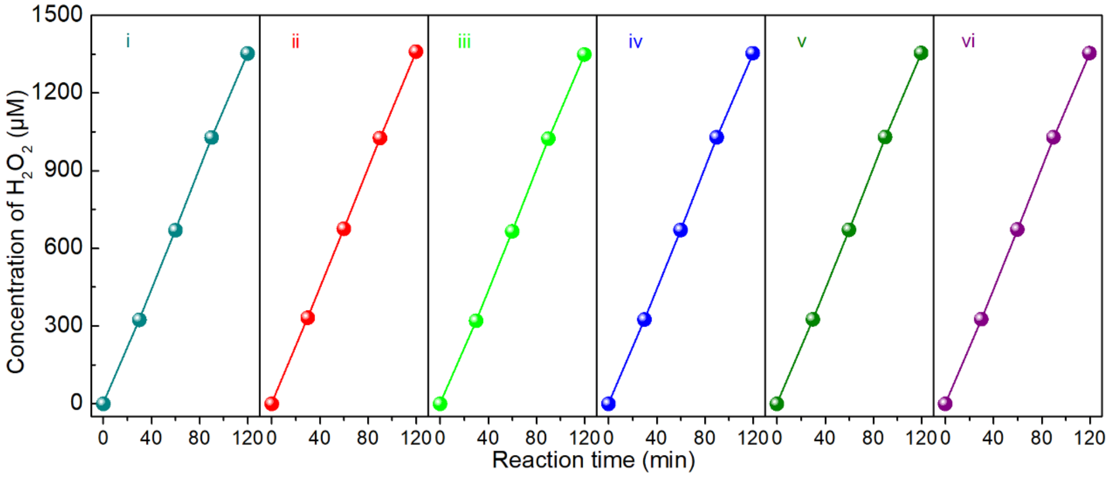

Figure S15. Six sequential recycling tests for the catalytic H_2O_2 production tests with $BaTiO_3$:Nb/C in an ethanol-water solvent under co-irradiation with ultrasound (40 kHz, 150 W) and visible light ($\lambda > 420$ nm, 100 mW cm^{-2}).

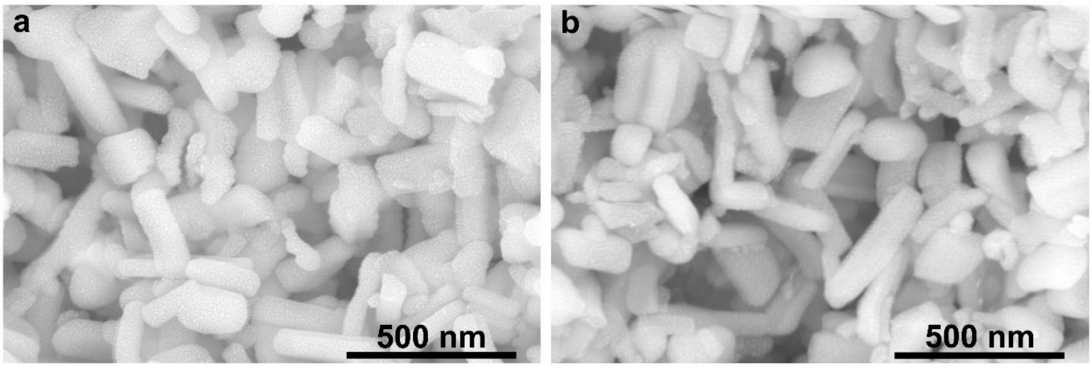

Figure S16. SEM images of $BaTiO_3$:Nb/C a) before and b) after six cycle used for H_2O_2 production under co-irradiation with ultrasound (40 kHz, 150 W) and visible light ($\lambda > 420$ nm, 100 mW cm^{-2}).

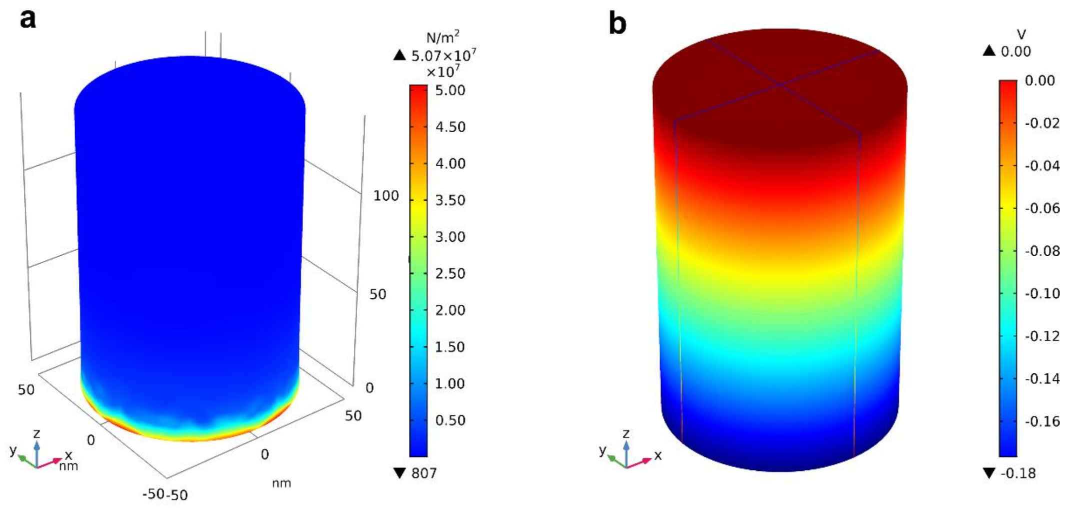

Figure S17. Finite element method simulation for the a) strain and b) piezopotential distribution on the surface of $BaTiO_3$ nanorod with the cavitation pressure of 10^8 Pa.

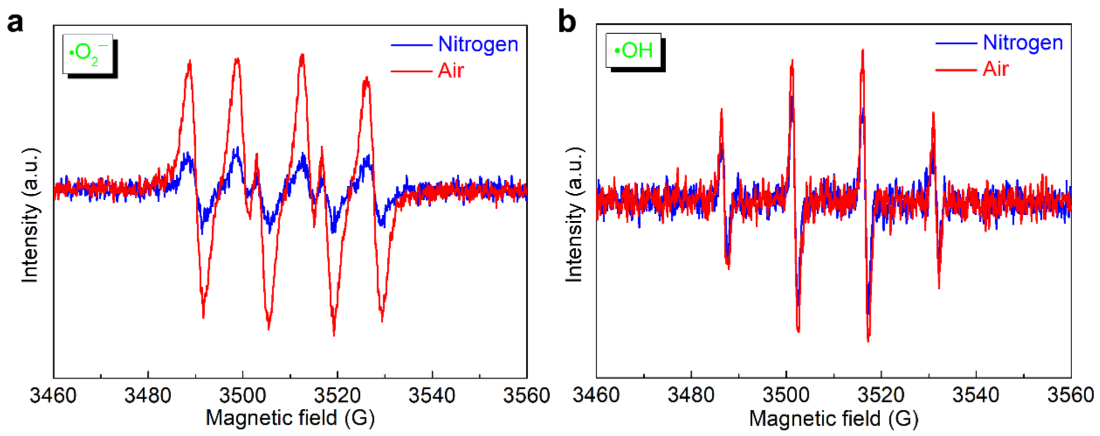

Figure S18. Electron spin resonance (ESR) spectra of $BaTiO_3$:Nb/C under co-irradiation with ultrasound (40 kHz, 150 W) and visible light (λ > 420 nm, 100 mW cm^{-2}) for three minutes in the presence of trapping reagent a) BMPO (5-tert-butoxycarbonyl-5-methyl-1-pyrroline N-oxide) or b) DMPO (5,5-dimethyl-1-pyrroline N-oxide) with air or nitrogen purging. We can clearly observe the characteristic signal of BMPO trapped $•O_2^-$ radicals and the signal intensity in air was much higher than that in nitrogen conditions, suggesting that the $•O_2^-$ radicals mainly originated from the O_2 reduction by electrons. The BMPO-$•O_2^-$ signal in nitrogen purged solvents indicated that the production of $•O_2^-$ radicals might be caused by residual O_2 in the solvents, or produced by series of oxidation/dimerization of H_2O. Simultaneously, an archetypal 1:2:2:1 quartet signal assigned to DMPO-•OH was observed and the signal intensity in nitrogen was near to in air conditions, indicating that the H_2O/OH^- had been oxidized by holes.

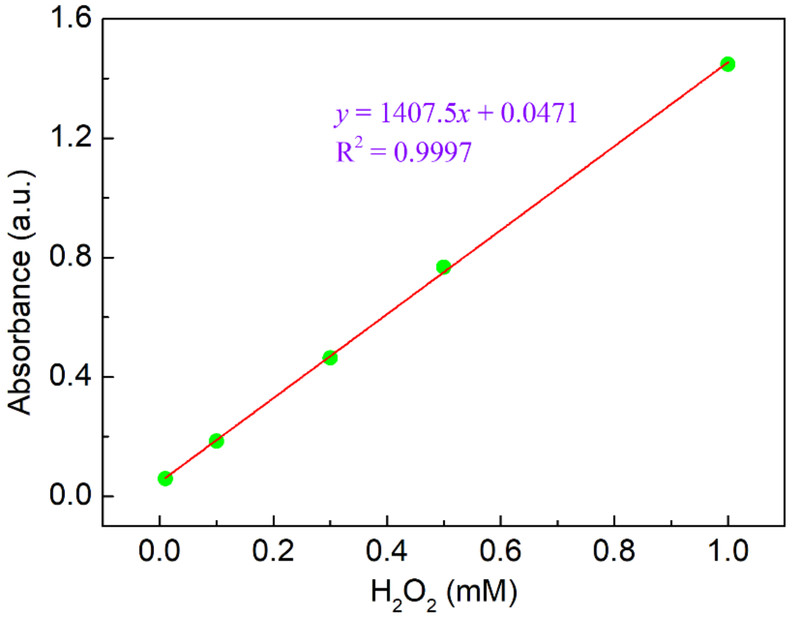

Figure S19. The calibration curve between the absorbance of a solution containing I_3^- ions and concentration of H_2O_2. The result of testing goodness of fit (R^2 = 0.9997) proves the model's applicability in the quantification of H_2O_2.

Table S1. Comparison of H_2O_2 production with representative catalysts

No.	Catalyst	Reaction type	Reaction conditions	Reaction rate	Reference
1	zinc polyphthalocyanine decorated on boron-doped carbon nitride	Photocatalysis	10 mg catalysts, 20 mL of H_2O; $\lambda >$ 400 nm, 100 mW cm^{-2}	114 $\mu mol\ g^{-1}\ h^{-1}$	*Proc. Natl. Acad. Sci.* (2021)[1]
2	ultrathin g-C_3N_4	Piezophotocatalysis	2 mg catalysts, 100 mL H_2O containing 0.1 M glucose; ultrasond (40 kHz, 240 W), visible light ($\lambda \geq$ 420 nm)	4.63 $mmol\ g^{-1}\ h^{-1}$	*Adv. Mater.* (2021)[2]
3	N,N,N′,N′-tetrakis(4-aminophenyl)-1,4-phenylenediamine (TAPD)-$(Me)_2$ and TAPD-$(OMe)_2$	Photocatalysis	20 mg catalysts, 420 < λ < 700 nm), 5.46 W m^{-2}	97 ± 10 and 91 ± 10 $\mu mol\ g^{-1}\ h^{-1}$	*J. Am. Chem. Soc.* (2020)[3]
4	$SnO_{2-x}/BiVO_4$ photoelectrode	Photoelectrochemical catalysis	1 M $NaHCO_3$ aqueous solution at pH 8.3; 300 W Xe arc lamp with an AM 1.5 filter, 100 mW cm^{-2}	0.83 $\mu mol\ min^{-1}\ cm^{-2}$	*J. Am. Chem. Soc.* (2020)[4]
5	Mo modified-$BiVO_4$ photoanode and	Photoelectrochemical	1 M	0.66	*Energy*

	anthraquinone-2-carboxylic acid-modified carbon cathode	catalysis	$NaHCO_3$ aqueous solution; AM 1.5G light, 100 mW cm^{-2}	μmol min^{-1} cm^{-2}	*Environ. Sci.* (2020)[5]
6	Bi_4NbO_8Br nanoplates	Piezophotocatalysis	50 mg catalysts, 100 ml H_2O_2 containing 10 ml ethanol; 300 W Xe lamp (λ > 420 nm), ultrasound (40 kHz, 240 W)	792 μmol g^{-1} h^{-1}	*Adv. Funct. Mater.* (2019)[6]
7	$BaTiO_3$:Nb/carbon dots	Piezophotocatalysis	15 mg catalysts, 30 mL of H_2O containing 3 ml ethanol; λ > 420 nm, 100 mW cm^{-2}, ultrasound (40 kHz, 1500 W)	1357 μmol g^{-1} h^{-1}	**This work**

Table S2. Apparent quantum yield and solar-to-chemical conversion efficiency of samples in different reaction conditions

Efficiency / Sample	**Φ_{AQY}/%**[a]		**SCC/%**[b]	
	Photocatalysis	Piezophotocatalysis	Photocatalysis	Piezophotocatalysis
C	0	0	0	0
$BaTiO_3$	6.7×10^{-4}	2.1×10^{-3}	3.7×10^{-3}	1.2×10^{-2}
$BaTiO_3$:Nb	1.0×10^{-3}	1.8×10^{-2}	5.5×10^{-3}	1.0×10^{-1}

$BaTiO_3$/C	3.6×10^{-3}	3.9×10^{-3}	2.0×10^{-2}	2.1×10^{-2}
$BaTiO_3$:Nb/C	4.4×10^{-3}	2.6×10^{-2}	2.4×10^{-2}	1.4×10^{-1}
$BaTiO_3$:Nb-C	1.2×10^{-3}	1.1×10^{-2}	6.4×10^{-3}	6.2×10^{-2}

[a] The apparent quantum yields (Φ_{AQY}) were determined by monochromated light irradiation at λ = 420 nm within 120 min. The selected ultrasonic power and frequency were 150 W and 40 kHz, respectively.

[b] The solar-to-chemical conversion efficiencies (SCCs) were determined under AM1.5G simulated light irradiation at wavelength of λ > 420 nm after 120 min of reaction. The selected ultrasonic power and frequency were 150 W and 40 kHz, respectively.

References

[1] Y.-X. Ye, J. Pan, F. Xie, L. Gong, S. Huang, Z. Ke, F. Zhu, J. Xu, G. Ouyang, *Proc. Natl. Acad. Sci.* **2021**, *118*, 2103964118.

[2] C. Hu, F. Chen, Y. Wang, N. Tian, T. Ma, Y. Zhang, H. Huang, *Adv. Mater.* **2021**, 2101751.

[3] C. Krishnaraj, H. Sekhar Jena, L. Bourda, A. Laemont, P. Pachfule, J. Roeser, C. V. Chandran, S. Borgmans, S. M. J. Rogge, K. Leus, C. V. Stevens, J. A. Martens, V. Van Speybroeck, E. Breynaert, A. Thomas, P. Van Der Voort, *J. Am. Chem. Soc.* **2020**, *142*, 20107-20116.

[4] K. Zhang, J. Liu, L. Wang, B. Jin, X. Yang, S. Zhang, J. H. Park, *J. Am. Chem. Soc.* **2020**, *142*, 8641-8648.

[5] T. H. Jeon, H. Kim, H.-i. Kim, W. Choi, *Energy Environ. Sci.* **2020**, *13*, 1730-1742.

[6] C. Hu, H. Huang, F. Chen, Y. Zhang, H. Yu, T. Ma, *Adv. Funct. Mater.* **2020**, *30*, 1908168.
